# Efficacy and safety of stem cell therapy vs. standard of care in patients diagnosed with acute respiratory distress syndrome: an updated systematic review and meta-analysis of randomized controlled trials

**DOI:** 10.3389/fmed.2025.1674720

**Published:** 2026-01-14

**Authors:** Jerin Jose Cherian, Saibal Das, Bhavani Shankara Bagepally, Madhavi Eerike, Sayan Nath, Alka Khadwal

**Affiliations:** 1 Indian Council of Medical Research, New Delhi, India; 2 Department of Global Public Health, Karolinska Institutet, Stockholm, Sweden; 3 Academy of Scientific and Innovative Research (An Institution of National Importance Established by an Act of Parliament), Ghaziabad, India; 4 Indian Council of Medical Research-National Institute for Research in Bacterial Infections, Kolkata, India; 5 Indian Council of Medical Research-National Centre for Disease Informatics and Research, Bengaluru, India; 6 Department of Pharmacology, All India Institute of Medical Sciences, Bibinagar, Telangana, India; 7 Department of Intensive Care, Cambridge University Hospitals NHS Foundation Trust, Cambridge, United Kingdom; 8 Department of Clinical Haematology and Medical Oncology, Post Graduate Institute of Medical Education and Research, Chandigarh, India

**Keywords:** acute respiratory distress syndrome (ARDS), anti-inflammatory drugs, coronavirus disease 2019 (COVID-19), mortality, stem cell

## Abstract

**Objectives:**

This systematic review and meta-analysis aimed to evaluate the efficacy and safety of stem cell therapies as compared to the standard of care (SOC) in patients with acute respiratory distress syndrome (ARDS).

**Methods:**

Search of PubMed, Embase, Cochrane CENTRAL, and Web of Science databases for randomized controlled trials was performed. The protocol was registered in PROSPERO (ID: CRD42023467612). The primary outcomes were all-cause mortality on day 28 and serious adverse events. Risk ratios (RR) and mean differences were pooled using Stata software version 17.0. Quality of the evidence was assessed by GRADE approach.

**Results:**

Out of 5,537 articles screened, 17 were included. Treatment with stem cells led to no significant difference in the risk of 28-day mortality [RR, 0.809 (95% CI: 0.651–1.005), *p* = 0.06; *I*^2^ = 0%] or the risk of serious adverse events [RR, 0.94 (95% CI: 0.80–1.12), *p* = 0.36; *I*^2^= 8.58%] as compared to treatment with SOC. Additionally, no significant differences were observed in the duration of hospitalization, the number of ventilator-free days till day 28, 60-day all-cause mortality, intensive care unit (ICU)-free days till day 28, change in quality-of-life (QoL) score, and the duration of ICU stay, PaO_2_/FiO_2_ ratio, change in SOFA score, and change in serum interleukin 6 and 8 levels. The GRADE of evidence was low or very low for the critical outcomes.

**Conclusion:**

There was no significant improvement in critical outcomes following stem cell therapy as compared to the SOC in ARDS. The certainty of evidence was low to very low, indicating limited confidence in the findings.

**Systematic Trial registration:**

PROSPERO (ID: CRD42023467612)

## 1 Introduction

Acute respiratory distress syndrome (ARDS) is a severe respiratory condition marked by hypoxemia and stiff lungs, often necessitating invasive mechanical ventilation (1). Approximately 10% of all patients admitted to the intensive care unit (ICU) and 23% of those on mechanical ventilation fulfilled the criteria for ARDS, leading to an annual ICU incidence rate of 5.5 cases per bed (2). The common risk factors of ARDS include severe pneumonia, sepsis, trauma, aspiration of gastric contents, etc. (3). Despite decades of clinical studies, pharmacological treatments, such as glucocorticoids, pulmonary surfactants, inhalational nitric oxide, antioxidants, anti-inflammatory drugs, and protease inhibitors, have not demonstrated efficacy, and supportive therapies remain the mainstay in the management of ARDS. The weighted average mortality rate across 102 studies published from 2009 to 2019 was 39.4% [95% confidence interval (CI): 37.0%−41.8%] (4).

Stem cell therapy has gained attention as a promising treatment option for ARDS. Different types of cells, including embryonic stem cells, induced pluripotent stem cells, and mesenchymal stromal stem cells (MSC), are being explored for their potential benefits in ARDS (5). These stem cells possess unique regenerative and immunomodulatory properties that promote lung tissue repair and modulate the exaggerated inflammatory response seen in ARDS (6). Stem cell-based therapies for ARDS operate through various mechanisms, such as migrating to the site of lung injury, modulating immune and inflammatory responses, exerting paracrine effects through cytokines, releasing beneficial exosomes, and reducing pulmonary fibrosis. Additionally, studies have demonstrated the safety of stem cell use in ARDS (6–8). After the emergence of the Coronavirus Disease 2019 (COVID-19) stem cell therapy was widely used in patients with ARDS. However, the evidence is not consistent in this regard. Most trials have small sample sizes, high heterogeneity, and low-certainty evidence. Additionally, many studies focus on COVID-19-related ARDS, limiting generalizability to other etiologies. This systematic review and meta-analysis aimed to evaluate the efficacy and safety of stem cell therapies including stem cells, stem cell-derived extracellular vesicles (EVs), and related products, in patients with ARDS, including those with COVID-19-related ARDS.

## 2 Methods

### 2.1 Eligibility criteria

Randomized controlled trials that enrolled patients of all age groups with a diagnosis of ARDS, regardless of the severity, ventilation requirements, or underlying etiology (infectious, non-infectious, COVID-19, pulmonary, or extra-pulmonary causes) were included. The trials included in the review involved the administration of any form of stem cell therapy or stem cell-derived products via any route. The stem cell therapy or products were administered for varying durations following the ARDS diagnosis, and they were compared to standard of care (SOC) or placebo. Studies that did not provide data on critical outcomes were excluded. Furthermore, studies were excluded if they lacked sufficient data, had inaccessible full-text articles, review articles, non-human research, duplicate publications, or multiple reports from the same study. Our review did not include conference abstracts, gray literature, or unpublished studies, which may increase the risk of publication bias because small or negative trials are less likely to be formally published. Additionally, although we attempted to obtain inaccessible full texts, their exclusion may have reduced the completeness of the evidence base.

### 2.2 Information sources

Databases, such as PubMed, Web of Science, Cochrane CENTRAL, and Embase, were searched from inception to October 2024. Search terms were tailored for different bibliographic databases, incorporating database-specific filters, with no restrictions on language. The PICO search strategy is enumerated in Supplementary Table S1.

### 2.3 Study selection

Using the search strategy, two independent authors (ME and SD) screened the titles and abstracts of relevant studies, without language restrictions, to identify those meeting the eligibility criteria. All disagreements were resolved with a third author (JJC). To assess suitability, the authors retrieved the study abstracts and, when necessary, the full-text articles. The web-based Rayyan software (https://www.rayyan.ai/) was utilized for this process.

### 2.4 Data extraction

Data were independently extracted by two authors (ME and JJC) using a standardized spreadsheet. The extracted information included the general characteristics of the studies, details of the population, intervention, comparison group, and outcomes relevant to the study objectives. No assumptions or simplifications were applied during the extraction process. Attrition, including withdrawals, loss to follow-up, and dropouts, was thoroughly examined, along with a critical evaluation of missing data and any methods of data imputation.

### 2.5 Risk of bias assessment

The assessment of the risk of bias for the critical outcomes in selected randomized controlled trials was done by using the revised Cochrane risk-of-bias 2 tool (9) by two independent authors (ME and SD). All disagreements were resolved with a third author (JJC).

### 2.6 Outcomes

The main outcomes assessed were all-cause mortality at 28 days and the occurrence of serious adverse events (SAEs). The duration of hospitalization, number of ventilator-free days till day 28, 60-day all-cause mortality, ICU-free days till day 28, change in quality-of-life (QoL) score, and duration of ICU stay, PaO_2_/FiO_2_ [the ratio of partial pressure of oxygen (PaO_2_) in arterial blood to the fraction of inspired oxygen (FiO_2_)], sequential organ failure assessment (SOFA) score change from day 1 to 14, and change in serum interleukin 6 and 8 levels were the secondary outcomes.

### 2.7 Data synthesis

Descriptive statistics were applied in cases of insufficient data. The risk ratio (RR) for dichotomous variables was computed for each study and subsequently pooled using a random-effects model (DerSimonian–Laird). Similarly, for continuous variables, mean differences with 95% confidence intervals (CI) were pooled and represented as a forest plot using a random-effects model (DerSimonian–Laird) or a fixed-effects model (e.g., inverse variance or Mantel–Haenszel) when heterogeneity was low (*I*^2^ < 50%). Sub-group analyses were performed according to the etiology of ARDS, trial phase, trial duration, the type of stem cell or derived product, and the source of stem cells used for treatment. Heterogeneity was assessed using a funnel plot, the Cochrane *Q* test, and *I*^2^ statistics. It was further evaluated with the χ^2^ test on n-1 degrees of freedom, using a 5% alpha error for statistical significance, and the *I*^2^ test. *I*^2^ values were interpreted as follows: < 25% indicated low heterogeneity, 25%−50% represented moderate heterogeneity, and >50% indicated high heterogeneity. The heterogeneity of treatment effect was considered present if the *p*-value from the Cochrane *Q* test was < 0.05 and the *I*^2^ value was >25%. Statistical analyses were conducted using Stata version 17.0 (Stata Corp, Texas). For all tests, a two-sided *p*-value of < 0.05 was deemed statistically significant, except for heterogeneity or subgroup analyses, where a one-sided *p*-value of < 0.1 was considered significant.

### 2.8 Sensitivity analysis and publication bias representation

Leave-one-out method was used for sensitivity analysis. A funnel plot was utilized to evaluate potential small-study effects and publication bias, provided there were at least ten studies available for analysis. The L'Abbé graph was plotted with the occurrence of an outcome or event to understand the distribution of studies based on the effect measures, to identify outliers, and to explore heterogeneities.

### 2.9 Certainty assessment

The GRADE approach (Grading of Recommendations Assessment, Development, and Evaluation) was employed to evaluate the quality of the evidence for the outcomes included in the pooled analyses (10, 11).

### 2.10 Ethics and registration

The study commenced following the receipt of an “exemption from review” from the Institutional Ethics Committee. The study protocol was registered with the International Prospective Register of Systematic Reviews (PROSPERO ID: CRD42023467612).

## 3 Results

**Figure 1:**
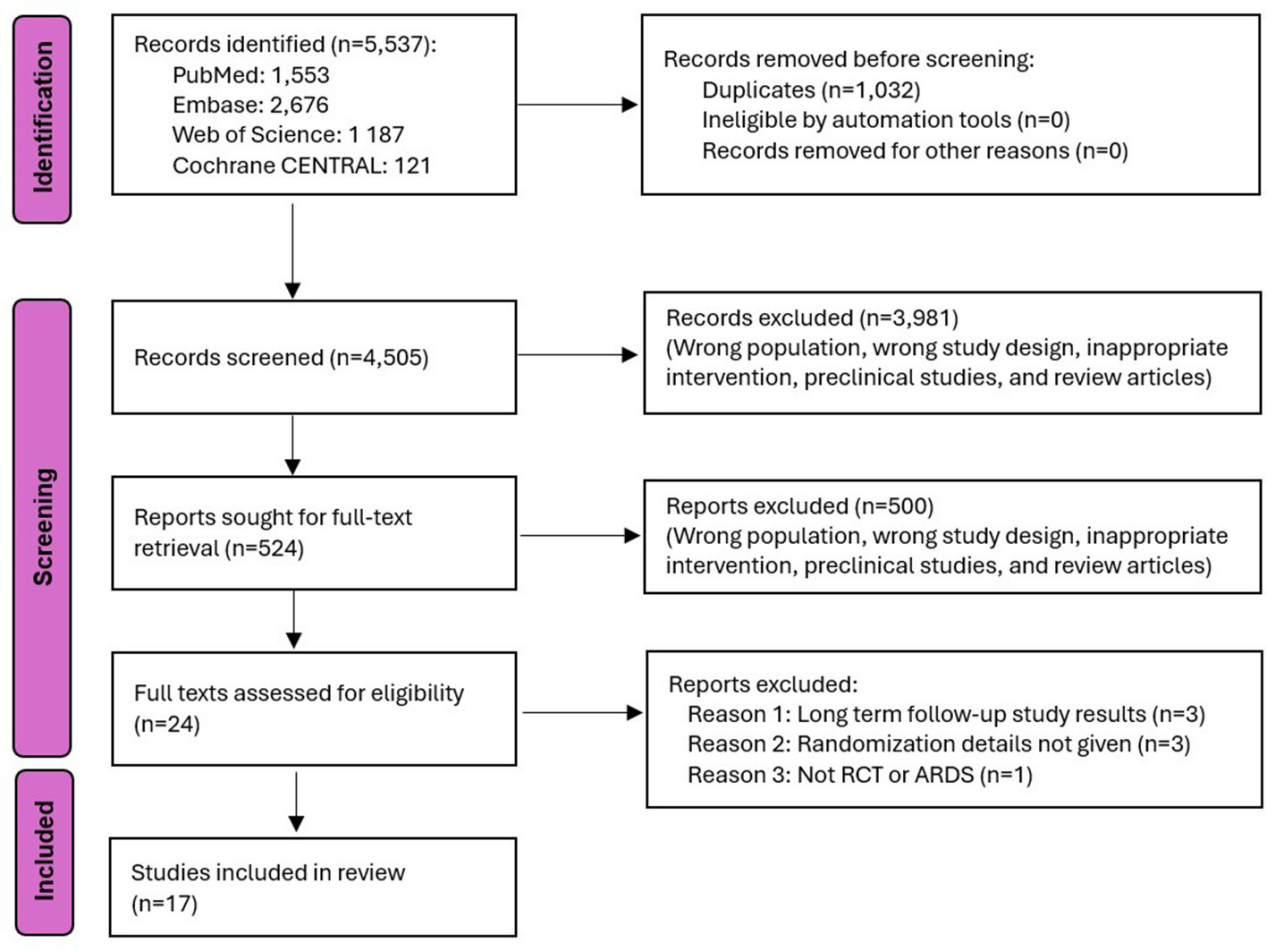
Study flow chart. ARDS, acute respiratory distress syndrome; RCT, randomized controlled trials.

A total of 5,537 studies were screened, and ultimately, 17 studies (12–28) (*n* = 829) were included in the analysis (Figure 1). Details regarding the characteristics of the individual studies are provided in Table 1. The risk of bias assessment for each study included in the analysis is shown in Table 2, highlighting the specific biases associated with each study. Of the included studies, one was identified as having a high risk of bias, while five studies were classified as having a moderate risk of bias.

**Table 1:** Baseline characteristics of the included studies.

**Author, year**	**Study population**	*N* ^*^	Age (years)^*^	Male/female ratio^*^	**Primary outcome**	**Intervention**	**Comparator**	**Study duration (days)**	**Key conclusion regarding stem-cell intervention**
Pochon, 2023, France	COVID-19-related moderate to severe ARDS	15:15	61 ± 12.59 vs. 66 ± 6.67	13/2 vs. 7/8	Proportion of patients with a PaO_2_/FiO_2_ ratio of >200 mm Hg on day 10	1 × 10^6^ MSC/kg on day 1, 0.5 × 10^6^ MSC/kg on day 4 and 6	Placebo	5	Treatment was safe No increased proportion of patients with a PaO_2_/FiO_2_ ratio of >200 mm Hg on day 10 after treatment 90-day mortality did not improve
Lightner, 2023, USA	COVID-19-related moderate to severe ARDS	68:34	59.4 vs. 58.5 ± 11.76	43/25 vs. 24/10	Mortality rate within 60 days	Exo Flo 15 ml/Exo Flo 10 ml (1.2 trillion bone marrow-MSC-derived extracellular vesicles per dose)	Placebo	60	Two doses of ExoFlo 15 ml significantly reduced mortality Intervention was safe
Bellingan, 2022, USA and UK	Moderate-to-severe ARDS	20:10	51 ± 14 59 ± 18	13/7 vs. 6/4	Safety and tolerability	900 million cells diluted into 300 ml of Plasmalyte-A	Placebo-	28	Multipotent adult progenitor cells at doses up to 900 million cells were safe and well-tolerated
Ichikado, 2023, Japan	COVID-19-related ARDS	20:10	69.2 ± 13.2 vs. 66.5 ± 10.0	16/4 vs. 10/0	Survival free from mechanical ventilation at 28 days	9.0 × 10^8^ cells of bone marrow-derived multi-potent adult progenitor cells i.v. infusion	Standard of care	180	No significant improvement in ventilator-free days Improved survival Intervention was well-tolerated
Zarrabi M, 2023, Iran	COVID-19-related ARDS	19:24	49.05 vs. 49.4 ± 11.7	15/4 vs. 16/6	Safety	100 × 10^6^ MSC i.v. infusion and one dose of MSC-derived EVs (200 × 10^6^) inhalation	Placebo	28	Reduction of serum level of inflammatory markers No SAEs
Rebelatto, 2022, Brazil	COVID-19-related ARDS	11:6	53 ± 15.3 vs. 61.7 ± 9.7	8/3 vs. 4/2	Safety	5 × 10^5^ cells/kg UC-MSCs i.v. infusion	Placebo	120	Safe and effective in the early and chronic stages
Fathi-Kazerooni, 2022, Iran	COVID-19-related ARDS	15:15	46.43 ± 11.91 vs. 53.67 ± 10.3	9/6 vs. 10/5	Safety	5 ml of menstrual blood-derived MSC i.v. infusion for 5 consecutive days for 60 min	Placebo	28	Reversal of hypoxia, immune reconstitution, and downregulation of cytokine storm No SAE
Gorman, 2023, UK	COVID-19-related moderate to severe ARDS	30:29	58.4 ± 9.2 vs. 58.4 ± 12.5	24/6 vs. 20/9	Safety and oxygenation index on day 7	ORBCEL-C (400 × 10^6^ CD362-enriched umbilical cord-derived MSCs in 200 ml Plasma-Lyte)	Placebo	90	Improved surrogates of pulmonary organ dysfunction No SAE
Zheng, 2014, China	ARDS	6:6	66.7 ± 20.4 vs. 69.8 ± 9.1	6/0 vs. 5/1	Safety	1 × 10^6^ cells/kg of allogeneic adipose-derived MSCs	Placebo	28	No SAE Inflammatory biomarker levels were reduced
Aghayan, 2022, Iran	COVID-19-related ARDS	10:10	62.3 vs. 58.4	6/4 vs. 8/2	Safety	Single dose of placenta-derived MSCs 1 × 10^6^ cells/kg	Standard treatment	28	No significant difference in the duration of hospitalization, oxygen saturation, and clinical and laboratory parameters No SAE
Dilogo, 2021, Indonesia	COVID-19-related severe ARDS	20:20	18�95 (overall)	15±5 vs. 15±5	Mortality rate and duration of ventilation	1 × 106 UC-MSCs/kg i.v. infusion	Placebo	15	Reduced mortality rate by modulating the immune system
Lanzoni, 2021, USA	COVID-19-related ARDS	12:12	58.58 ± 15.93 vs. 58.83 ± 11.61	5/7 vs. 8/4	Safety	100 ± 20 × 10^6^ UC-MSCs i.v. infusion	Placebo	28	Reduction in SAE Reduced mortality rate and faster time to recovery
Matthay, 2019, USA	COVID-19-related moderate to severe ARDS	40:20	55 ± 17 vs. 55 ± 20	23/17 vs. 10/10	Safety	10 × 10^6^ MSC/kg i.v. infusion	Placebo	60	Reduction in SAE No significant reduction in mortality
Bowdish, 2023, USA	COVID-19-related moderate to severe ARDS	112:110	61.8 ± 13 vs. 59.6 ± 13.8	79/33 vs. 75/35	All-cause mortality within 30 days	2 × 10^6^ MSC/kg i.v. infusion	Placebo	365	No significant reduction in 30-day mortality rate or 60-day ventilator-free days
Monsel, 2022. France	COVID-19-related moderate to severe ARDS	21:24	64 ± 10.4 vs. 63.2 ± 11.4	17/4 vs. 20/4	Change in PaO_2_/FiO_2_ ratio on day 7	3 × 10^6^ umbilical cord-MSCs/kg i.v. infusion	Placebo	28	No significant difference in the PaO_2_/FiO_2_ ratio on day 7 No SAE
Martínez-Muñoz. 2024, Spain	COVID-19-related moderate to severe ARDS	10:10	59.5 vs. 65.5	5/5 vs. 8/2	Change in PaO_2_/FiO_2_ ratio on day 7	1 × 10^6^MSC/kg i.v. infusion	Placebo	12 months	No significant difference in the PaO_2_/FiO_2_ ratio on day 7
Zamanian, 2024, Iran	COVID-19 and ARDS	21:24	54.24 ± 15.93 vs. 62.08 ± 16.66	17/4 vs. 17/7	Mortality rate	1.5–2 × 10^9^ extracellular vesicles/kg	Placebo	Till death or discharge	Treatment was safe and effective

^*^Intervention vs. comparator.

ARDS, acute respiratory distress syndrome; COVID-19, Coronavirus Disease 2019; i.v., intravenous; MSC, mesenchymal stem cells; PaO_2_/FiO_2_, partial pressure of oxygen in arterial blood to the fraction of inspired oxygen; SAE, serious adverse events; UK, United Kingdom; USA, United States of America.

**Table 2:** Risk of bias of each individual study according to the Cochrane risk of bias-2 tool for all-cause mortality for 28 days.

**Author, year**	**Bias arising from the randomization process**	**Bias due to deviations from intended interventions**	**Bias due to missing outcome data**	**Bias in measurement of the outcome**	**Bias in selection of the reported result**	**Overall bias**
Pochon, 2023	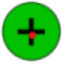	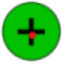	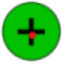	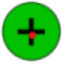	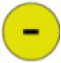	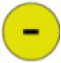
Lightner, 2023	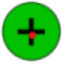	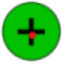	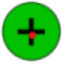	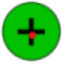	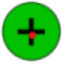	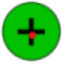
Bellingan, 2022	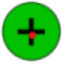	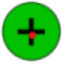	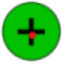	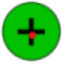	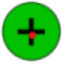	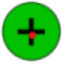
Ichikado, 2023	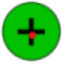	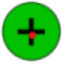	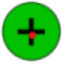	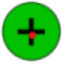	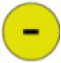	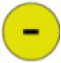
Zarrabi M, 2023	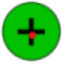	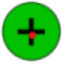	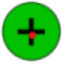	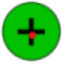	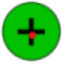	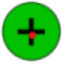
Rebelatto, 2022	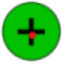	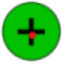	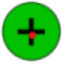	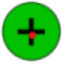	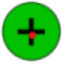	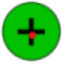
Fathi-Kazerooni, 2022	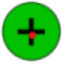	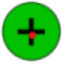	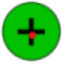	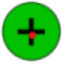	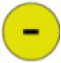	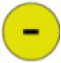
Gorman, 2023	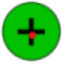	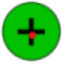	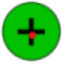	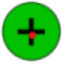	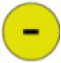	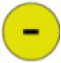
Zheng, 2014	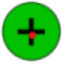	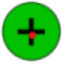	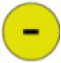	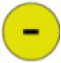	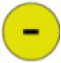	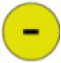
Aghayan, 2022	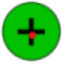	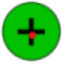	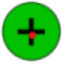	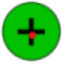	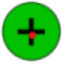	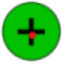
Dilogo, 2021	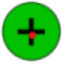	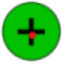	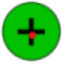	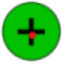	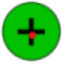	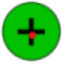
Lanzoni, 2021	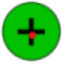	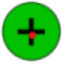	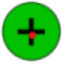	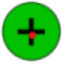	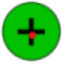	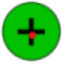
Matthay, 2019	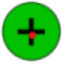	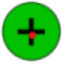	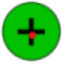	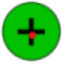	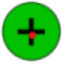	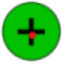
Bowdish, 2023	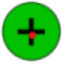	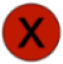	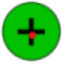	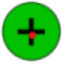	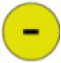	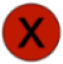
Monsel, 2022	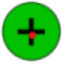	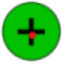	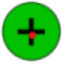	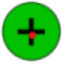	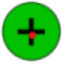	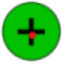
Pochon, 2023	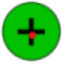	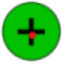	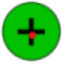	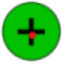	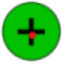	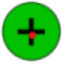
Lightner, 2023	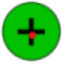	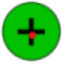	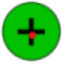	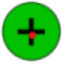	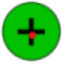	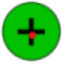

In this review, most of the studies included were phase 1/2 trials. The studies varied widely in sample sizes, ranging from 12 to 222 participants, with most involving small to moderate cohorts. The interventions primarily utilized MSCs derived from umbilical cord, bone marrow, or menstrual blood, administered through intravenous infusions. Dosing strategies ranged from single infusions (e.g., 1 × 10^6^ MSCs/kg) to repeated doses over several days, and a few studies explored EVsderived from stem cells. The route of administration of stem cell therapy was predominantly intravenous (i.v.) infusion, except for one study where the stem cell-derived product was delivered via inhalation. Among the 17 studies, mesenchymal stem cells were the primary focus in 12 studies; two studies investigated stem cell-derived products (EVs); one study evaluated a combination of stem cells and their derived products (MSC-derived EVs); and two studies used multipotent adult progenitor cells. The major sources of stem cells were either bone marrow or umbilical cord. The duration of follow-up in the majority of the studies was 28 days, while it was 90 days in one study.

**Figure 2:**
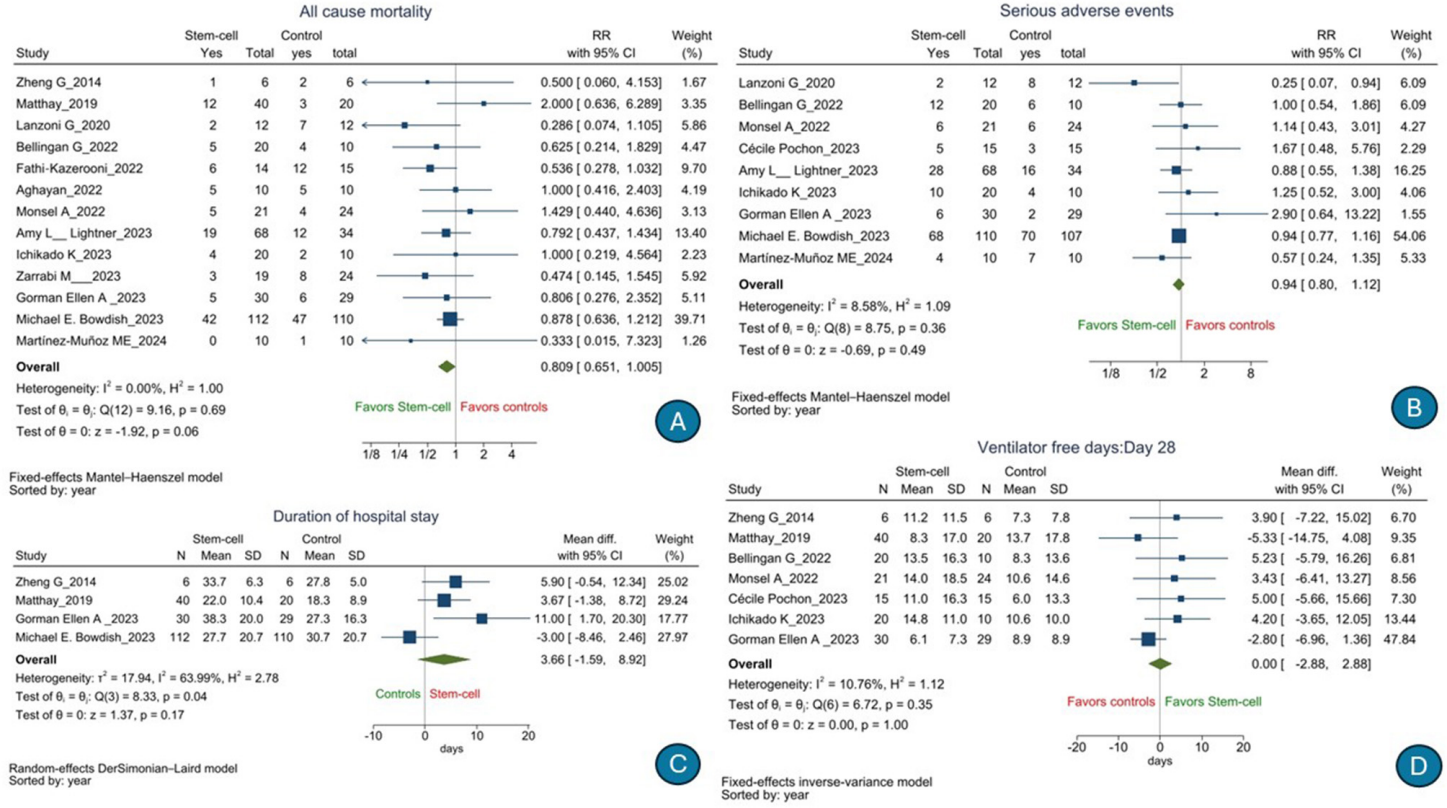
Effect of stem cell treatment as compared to the standard of care in patients with acute respiratory distress syndrome on 28-day all-cause mortality **(A)**, serious adverse events **(B)**, duration of hospitalization **(C)**, and number of ventilator-free days till day 28 **(D)**.

**Figure 3:**
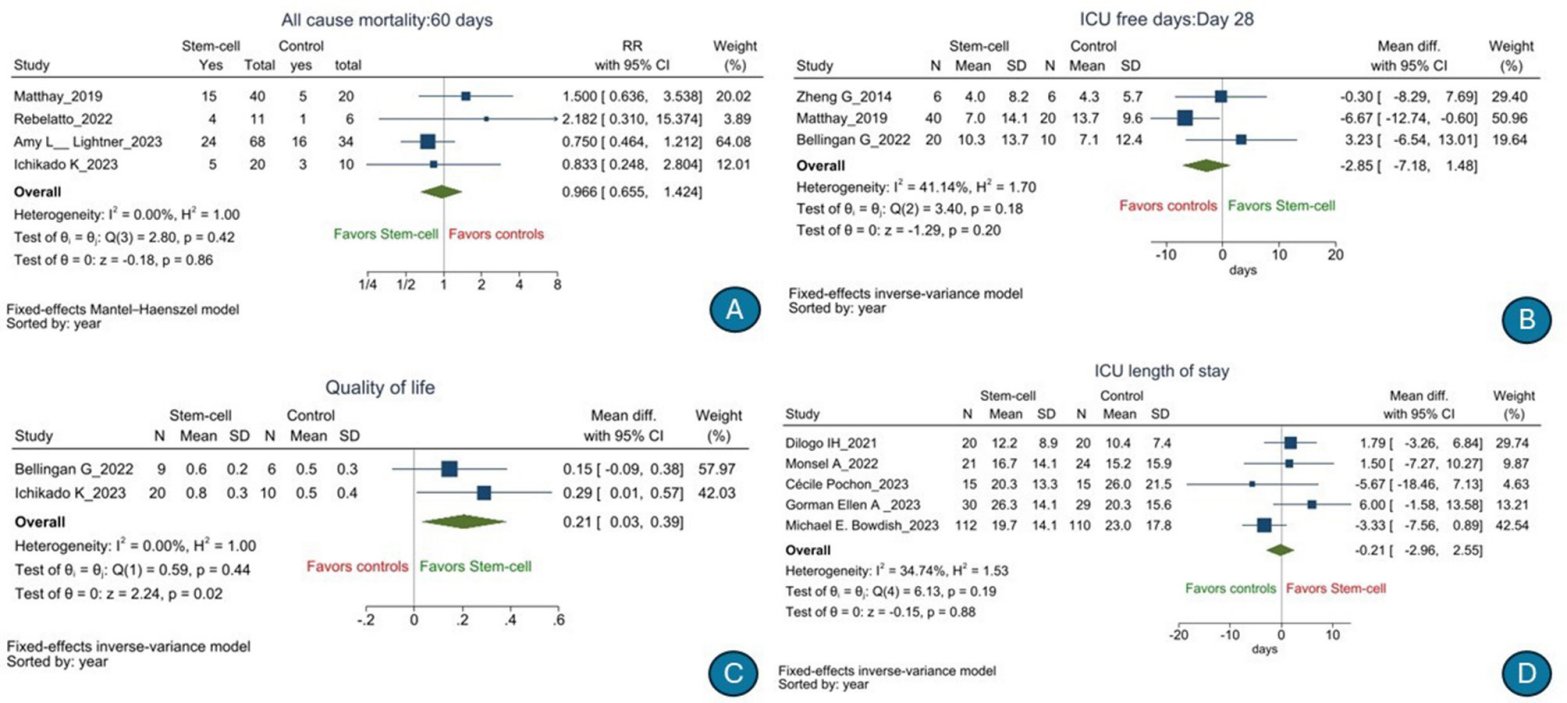
Effect of stem cell treatment as compared to the standard of care in patients with acute respiratory distress syndrome on 60-day all-cause mortality **(A)**, intensive care unit-free days till day 28 **(B)**, change in quality-of-life score **(C)**, and duration of intensive care unit stay **(D)**.

**Figure 4:**
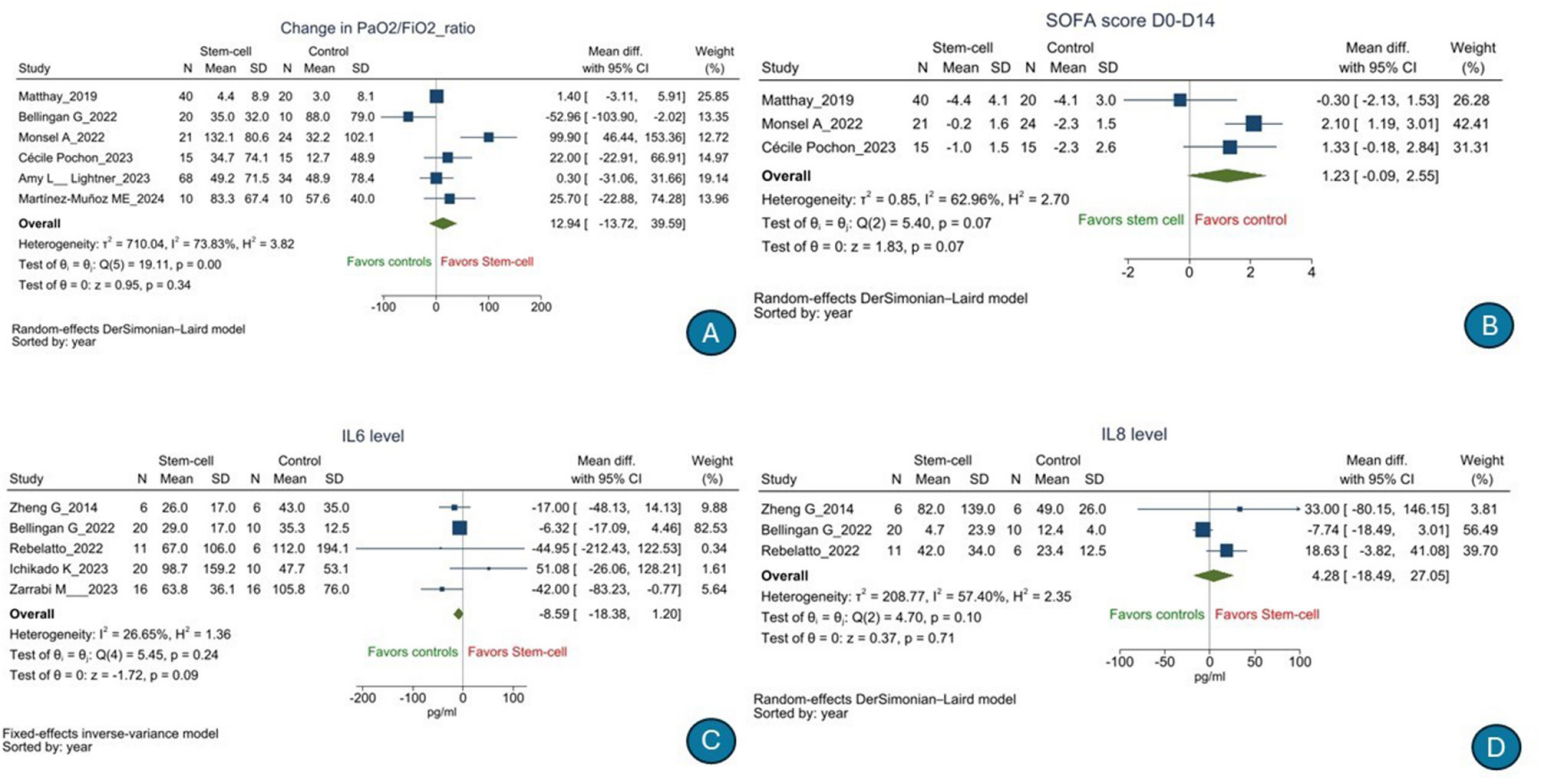
Effect of stem cell treatment as compared to the standard of care in patients with acute respiratory distress syndrome on PaO_2_/FiO_2_ ratio **(A)**, change in SOFA score from day 1 to 14 **(B)**, change in serum interleukin 6 levels **(C)**, and change in serum interleukin 8 levels **(D)**.

There was no significant difference in the risk of 28-day all-cause mortality [*n* = 676, RR, 0.809 (95% CI: 0.651–1.005), *p* = 0.06); *I*^2^ = 0%] (low GRADE evidence; Figure 2A) or serious adverse events [*n* = 484, RR, 0.94 (95% CI: 0.80–1.12), *p* = 0.36); *I*^2^ = 8.58%] (moderate GRADE evidence; Figure 2B) following stem cell therapy as compared to that following SOC. There was no significant difference in terms of the duration of hospitalization [*n* = 353, mean difference, 3.66 days (95% CI: −1.59 to 8.92), *p* = 0.17); *I*^2^ = 63.99%] (low GRADE evidence; Figure 2C), number of ventilator-free days till day 28 [*n* = 266, mean difference, 0.00 days (95% CI: −2.88 to 2.88), *p* = 1.00); *I*^2^ = 10.76%] (very low GRADE evidence; Figure 2D), 60-day all-cause mortality (low GRADE evidence) [RR, 0.966 (95% CI: 0.655–1.424), *p* = 0.86); *I*^2^ = 0%] (Figure 3A), ICU-free days till day 28 [*n* = 102, mean difference, −2.85 days (95% CI: −7.18 to 1.48), *p* = 0.20); *I*^2^ = 41.14%] (very low GRADE evidence; Figure 3B), duration of ICU stay [*n* = 396, mean difference, 0.21 days (95% CI: −2.96 to 2.55), *p* = 0.88); *I*^2^ = 34.74%] (very low GRADE evidence; Figure 3D), PaO_2_/FiO_2_ ratio [*n* = 267, mean difference, 12.94 (95% CI: −13.72 to 39.59), *p* = 0.34); *I*^2^ = 73.83%] (low GRADE evidence; Figure 4A), change in SOFA score from day 1 to 14 [*n* = 135, mean difference, 1.23 (95% CI: −0.09 to 2.55), *p* = 0.07); *I*^2^ = 62.96%] (very low GRADE evidence; Figure 4B), change in serum interleukin 6 level[mean difference *n* = 121, −8.59 pg/ml (95% CI: −18.38 to 1.20), *p* = 0.09); *I*^2^ = 26.65%] (very low GRADE evidence; Figure 4C), and change in serum interleukin 8 level [*n* = 59, mean difference, 4.28 pg/ml (95% CI: −18.49 to 27.05), *p* = 0.71); *I*^2^ = 57.40%] (very low GRADE evidence; Figure 4D) following stem cell therapy as compared to that following SOC.

However, there was some significant improvement in the change in quality-of-life score [*n* = 45, mean difference, 0.21 (95% CI: 0.03–0.39), *p* = 0.02); *I*^2^ = 0%] (low GRADE evidence; Figure 3C) following stem cell therapy as compared to that following SOC. Also, when post-treatment vs. pre-treatment values were compared, there was a significant difference in the IL-6 level [*n* = 121, mean difference, −89.56 pg/ml (95% CI: −161.64 to −17.49), *p* = 0.01); *I*^2^ = 88.43%] (Figure 3C) but not IL-8 level [*n* = 59, mean difference, −8.13 pg/ml (95% CI: −19.25 to 3.00), *p* = 0.15); *I*^2^ = 0%] (Figure 3D) among patients with ARDS who received stem cell therapy as compared to those who received the SOC (Supplementary Figures S41, S42). The results are consistent across various sub-groups. Publication bias could not be detected for any outcome. The summary of GRADE tables is listed in Table 3. The results of various sub-group analyses, sensitivity analyses, and publication bias detection (contour funnel plots and funnel plots) for all outcomes are presented in Supplementary Figures S1–S40 and S43–S46.

**Table 3:** Summary of GRADE tables.

**All-cause mortality (28 days)**											
676 (12 RCTs)	Serious^a^	Not serious	Not serious	Serious^b^	None	⊕⊕○○ Low^a, b^	112/304 (36.8%)	109/372 (29.3%)	RR 0.809 (0.651 to 1.005)	112/304 (36.8%)	68 fewer per 1,000 (from 127 fewer to 5 more)
**Ventilator-free days**											
266 (7 RCTs)	Serious^a^	Serious^b^	Not serious	Serious^c^	None	⊕○○○ Very low^a, b, c^	114	152	–	114	MD 0 (2.88 lower to 2.88 higher)
**ICU-free days**										
102 (3 RCTs)	Serious^a^	Serious^b^	Not serious	Serious^c^	None	⊕○○○ Very low^a, b, c^	36	66	–	36	MD 2.85 lower (7.18 lower to 1.48 higher)
**Change in PaO**_2_**: FiO**_2_ **ratio**										
267 (5 RCTs)	Serious^a^	Not serious	Not serious	Serious^c^	None	⊕⊕○○ Low^a, c^	103	164	–	103	MD 11.22 higher (19.39 lower to 41.84 higher)
**Duration of hospitalization**										
353 (4 RCTs)	Serious^a^	Not serious	Not serious	Serious^c^	None	⊕⊕○○ Low^a, c^	165	188	–	165	MD 3.66 higher (1.59 higher to 8.92 higher)
**Length of ICU stay**										
396 (5 RCTs)	Serious^a^	Serious^b^	Not serious	Serious^c^	None	⊕○○○ Very low^a, b, c^	198	198	–	198	MD 0.2 higher (3.5 lower to 3.9 higher)
**SOFA score**										
135 (3 RCTs)	Serious^a^	Serious^b^	Not serious	Serious^c^	None	⊕○○○ Very low^a, b, c^	59	76	–	59	MD 1.23 higher (0.09 lower to 2.55 higher)
**QoL**										
45 (2 RCTs)	Serious^a^	Not serious	Not serious	Serious^c^	None	⊕⊕○○ Low^a, c^	16	29	–	16	MD 0.21 higher (0.03 lower to 0.39 higher)
**IL-6 level**										
121 (5 RCTs)	Serious^a^	Serious^b^	Not serious	Serious^c^	None	⊕○○○ Very low^a, b, c^	48	73	–	48	MD 8.59 lower (18.38 lower to 1.2 higher)
**IL-8 level**										
59 (3 RCTs)	Serious^a^	Serious^b^	Not serious	Serious^c^	None	⊕○○○ Very low^a, b, c^	22	37	–	22	MD 4.28 higher (18.49 lower to 27.05 higher)
484 (8 RCTs)	Serious^a^	Not serious	Not serious	Not serious	None	⊕⊕⊕○ Moderate^a^	115/241 (47.7%)	137/243 (56.4%)	**RR 0.94** (0.80 to 1.12)	115/241 (47.7%)	**19 fewer per 1,000** (from 91 fewer to 67 more)
**All-cause mortality (60 days)**										
209 (4 RCTs)	Serious^a^	Not serious	Not serious	Serious^a, c^	None	⊕⊕○○ Low^a, c^	25/70 (35.7%)	48/139 (34.5%)	**RR 0.966** (0.655 to 1.424)	25/70 (35.7%)	**12 fewer per 1,000** (from 123 fewer to 151 more)

CI, confidence interval; MD, mean difference; RR, risk ratio.

^a^Studies are unpowered for determining efficacy.

^b^Studies showed diverse results.

^c^The 95% CI is crossing the line of no effect.

## 4 Discussion

From the 17 studies included in the analysis, we found that key clinical outcomes, such as 28-day all-cause mortality, serious adverse events, hospitalization duration, ventilator-free days, or oxygenation indices, were not statistically significant following stem cell therapy as compared to standard care (SOC). Some evidence suggested minor improvements in QoL scores and post-treatment interleukin-6 levels. However, the overall evidence quality was graded low, and significant heterogeneity was observed in some outcomes.

The reassuring lack of increased risk of SAEs compared to standard care suggests stem cell therapy is safe within the studied parameters. However, the prolonged duration of hospitalization in patients receiving stem cell therapy raises concerns about balancing potential survival benefits with resource and logistical implications. Notably, no randomized controlled trials or well-designed studies have been conducted within the Indian population context, further limiting applicability. By expanding the scope beyond previous reviews centered on safety and mortality, this analysis incorporated diverse outcomes, such as ventilator-free days, ICU-free days, inflammatory markers, and PaO_2_/FiO_2_ ratio changes, providing a more nuanced understanding of the therapy's impact. The lack of statistical significance for 28-day all-cause mortality and other outcomes highlights the need for cautious interpretation and further investigation into its role in ARDS management. The observed improvement in QoL, as measured using the EQ-5D-5L scale (range < 0–1), reached statistical significance, but its clinical importance remains unclear. No established minimal clinically important difference (MCID) exists for ARDS, and the magnitude of change is small compared to MCID values reported in other cohorts of critically ill patients. This finding must therefore be interpreted with caution.

This systematic review aligns with prior studies highlighting the limitations of stem cell therapy in ARDS, reinforcing findings that it does not significantly reduce mortality or serious adverse events. Our findings align with prior studies that highlight the limitations of stem cell therapy for ARDS. Although it was earlier demonstrated in a systematic review that MSC therapy is generally safe and significantly reduces mortality in ARDS patients (29), our updated review shows that it has no significant 28-day all-cause mortality benefits. In another study, it was found that MSCs did not significantly increase adverse events and showed moderate efficacy in reducing inflammatory markers like interleukin-6, suggesting their potential role as an adjunct therapy (30). Despite the limited improvement in some clinical outcomes, the immunomodulatory properties of MSCs, including their ability to regulate cytokine responses and reduce pulmonary inflammation, have been highlighted in various preclinical and clinical studies (31, 32). These properties offer a strong rationale for further investigation into optimal timing, dosing, and delivery routes to maximize therapeutic benefits. Previous authors emphasized the need for innovative approaches, such as combining MSCs with advanced supportive therapies like extracorporeal membrane oxygenation, to improve outcomes in severe ARDS cases (31). However, it is to be noted that MSCs and EVs are biologically different, while sharing overlapping immunomodulatory mechanisms; EVs are often considered MSC-derived therapeutic products. Because only a limited number of EV trials were available, we combined MSC and EV studies to preserve analytical power. These interventions are not clinically comparable, however, and analyses in larger datasets should consider these separately.

In earlier studies, MSCs have demonstrated the potential to mitigate this imbalance by reducing pro-inflammatory factors and enhancing anti-inflammatory responses. A previous study revealed that MSC treatment significantly reduced mortality in ARDS by alleviating inflammatory lung injury and promoting alveolar epithelial recovery. MSCs also significantly reduced inflammatory markers levels, such as CRP and IL-6, with IL-6 reduction being particularly noteworthy due to its critical role in ARDS-related immune hyperactivity and lung tissue invasion (33, 34). Lower baseline IL-6 levels were associated with improved survival, reflecting MSCs' potent anti-inflammatory effects. Although IL-6 levels decreased within the stem cell group, the between-group comparison was not statistically significant. The within-group reduction, therefore, cannot be interpreted as evidence of superior anti-inflammatory benefit, and conclusions must be based solely on between-group differences. While MSCs exhibited a trend toward reducing inflammatory markers, statistical significance was not observed (33–35). Clinical trials assessing MSC therapy in ARDS have shown that MSCs have been associated with a favorable trend in morality reduction, pulmonary function improvement, and cytokine correction (36). However, MSC's potential in ARDS lies in its ability to modulate inflammatory responses, as seen in reduced CRP and cytokine levels. Additional promising approaches include MSC-derived exosomes, which have shown safety and cytokine regulation in early studies. Challenges remain in optimizing MSC applications. Effective use may depend on correcting adverse microenvironments, such as high IL-6 and fibronectin levels, using antioxidants or anti-inflammatory agents (37, 38).

Compared to prior meta-analyses, our study expands the scope by including recent trials, assessing diverse clinical parameters, and addressing key gaps in methodological rigor and heterogeneity. However, there were some limitations to our study. First, there was significant clinical heterogeneity among the studies included, such as differences in ARDS etiology (COVID-19 vs. non-COVID), disease severity at baseline, timing of intervention, and follow-up duration. Second, sample sizes were small in many trials, which reduces statistical power and therefore can result in imprecision in the estimates of effects. Third, the types, sources, doses, and routes of administration of stem cells or EVs also differed considerably across studies, making comparison difficult. Similarly, there was considerable variability in the SOC that included either advanced supportive therapies or only basic management, which could influence comparative outcomes. Together, these factors introduce heterogeneity that may partly explain the inconsistent findings and contribute to the low or very low certainty of evidence. Fourth, some of the included studies had a moderate risk of bias. Fifth, most trials reported outcomes only up to 28–60 days, limiting conclusions about long-term efficacy and safety, which is particularly important for allogeneic cell-based therapies. Early (1–7 day) and long-term (1–2 year) responses were inconsistently reported across studies. Finally, not all outcomes of interest were reported in each study, which precluded inclusion in the pooled analyses. There were also some limitations in our review process. Data for all the outcomes of interest were not available, preventing their inclusion in the review or pooled analysis. Additionally, heterogeneity was high for some outcomes, and the quality of evidence (GRADE) for most outcomes was rated as low or very low.

## 5 Conclusion

This systematic review and meta-analysis, which included 17 studies out of 5,537 screened, found reduction in 28-day mortality or serious adverse events was not significant in stem cell therapy received ARDS patients compared to SOC. Secondary outcomes, including hospitalization duration, ICU-free days, ventilator-free days at day 28, quality-of-life scores, and changes in biomarkers, such as PaO_2_/FiO_2_ ratio, SOFA score, and serum interleukin levels, also showed no significant differences. The analysis indicates that the current evidence supporting the efficacy of stem cell therapy in ARDS is of low to very low certainty according to the GRADE assessment. These findings underscore the necessity for additional high-quality, large-scale randomized controlled trials to more thoroughly assess the clinical benefits and underlying mechanisms of stem cell therapy in the management of ARDS.

## Data Availability

The original contributions presented in the study are included in the article/Supplementary material, further inquiries can be directed to the corresponding authors.
