# Supplementary material for: Efficacy and safety of stem cell therapy vs. standard of care in patients diagnosed with acute respiratory distress syndrome: an updated systematic review and meta-analysis of randomized controlled trials
Source: Front Med (Lausanne). 2026 Jan 14;12:1674720. doi: 10.3389/fmed.2025.1674720 (PMC12847309; doi:10.3389/fmed.2025.1674720)
Supplement: Supplementary file 1 [file Data_Sheet_1.docx]

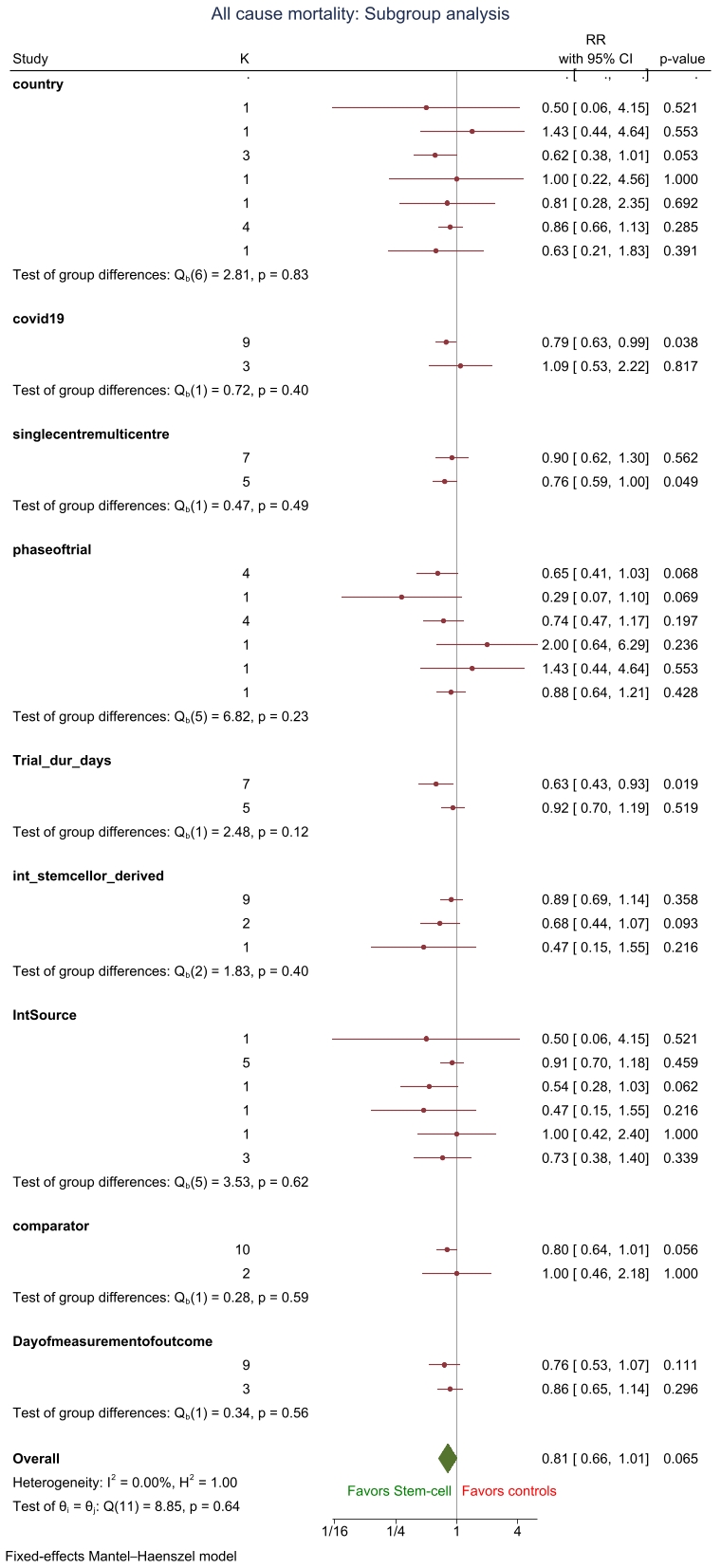


**Figure s1.** Sub-group analysis of the effect of stem cell treatment on 28-day all-cause mortality as compared to SOC in patients with ARDS.


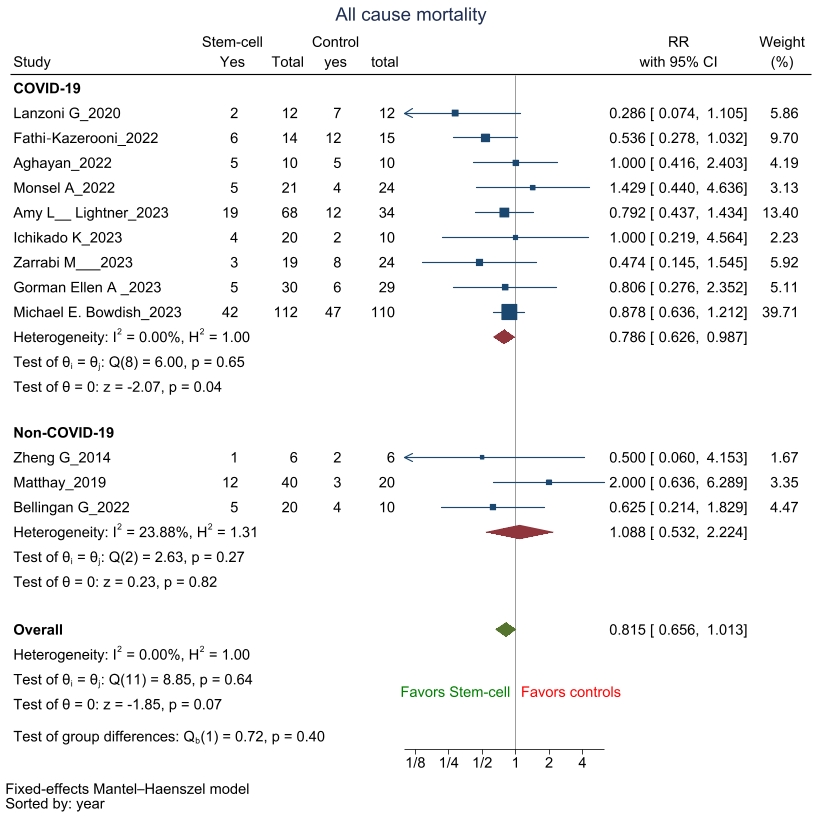


**Figure s2.** Sub-group analysis of the effect of stem cell treatment on 28-day all-cause mortality as compared to SOC in patients with ARDS based on etiology.


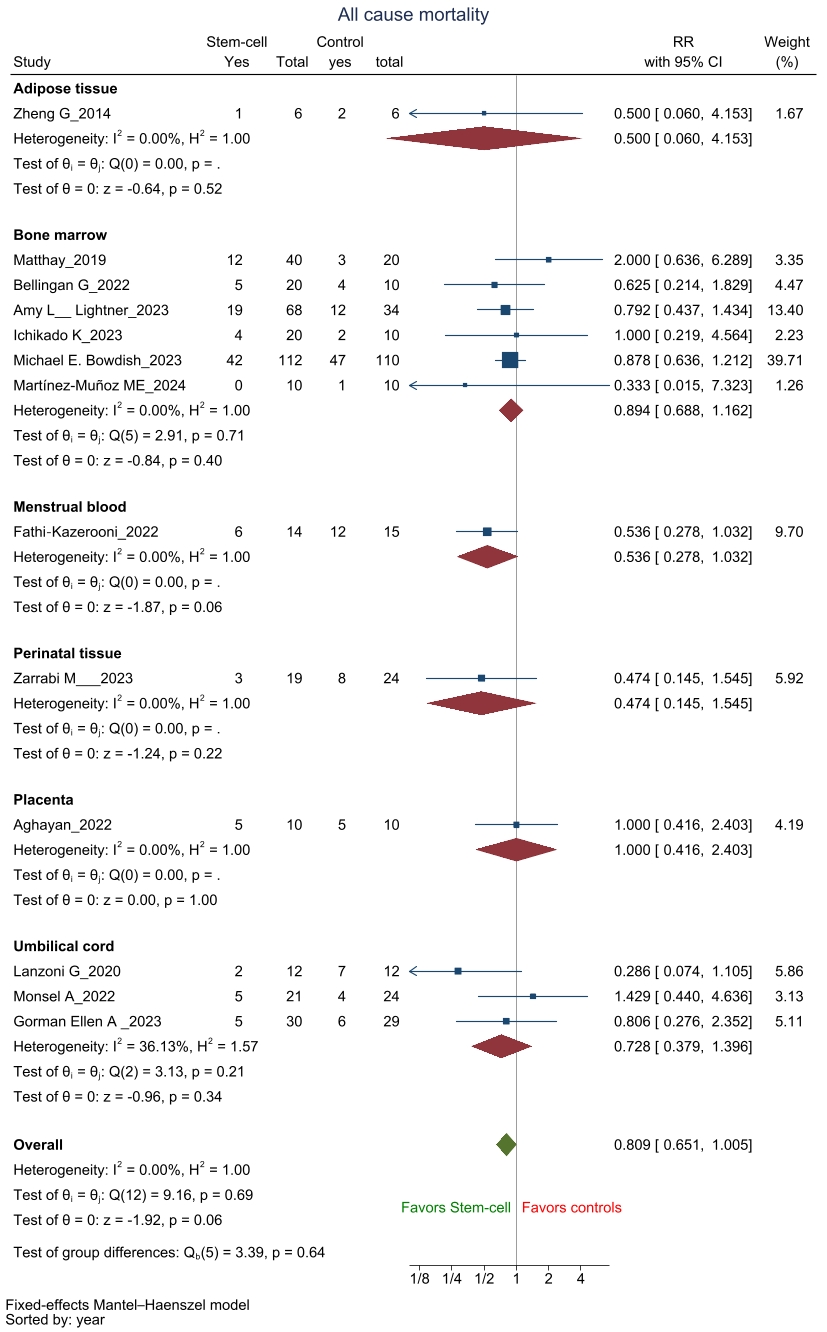


**Figure s3.** Sub-group analysis of the effect of stem cell treatment on 28-day all-cause mortality as compared to SOC in patients with ARDS based on source of stem cells.


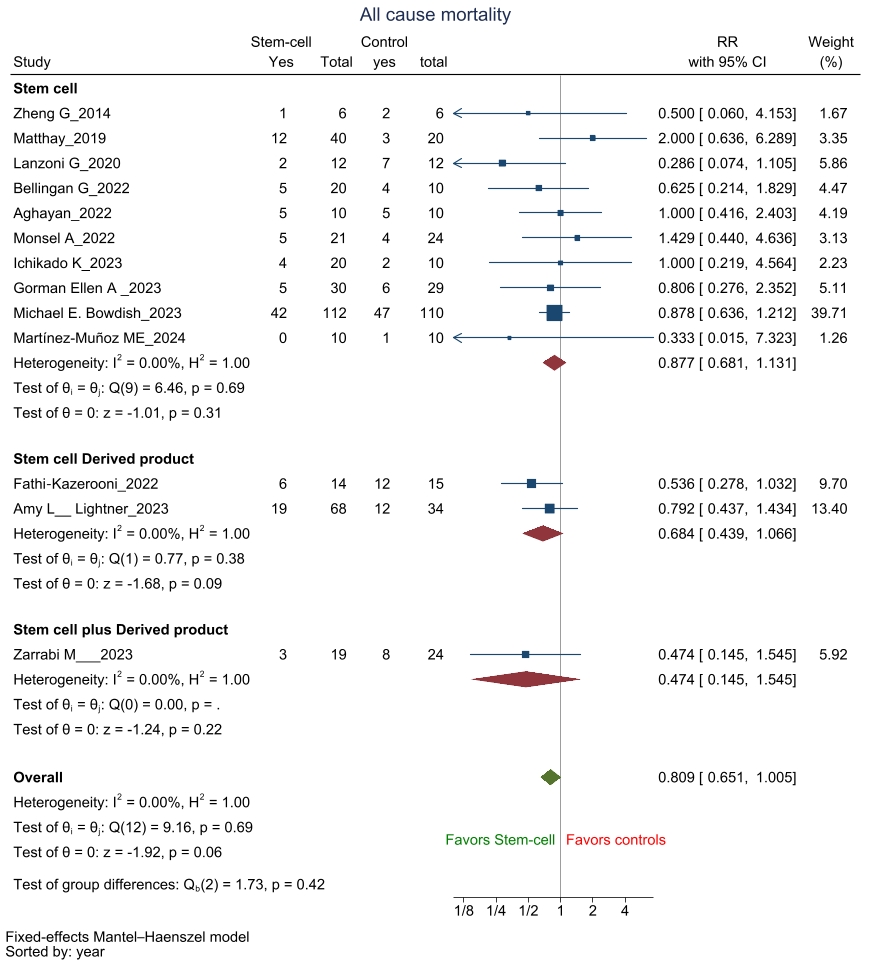


**Figure s4.** Sub-group analysis of the effect of stem cell treatment on 28-day all-cause mortality as compared to SOC in patients with ARDS based on type of stem cells.


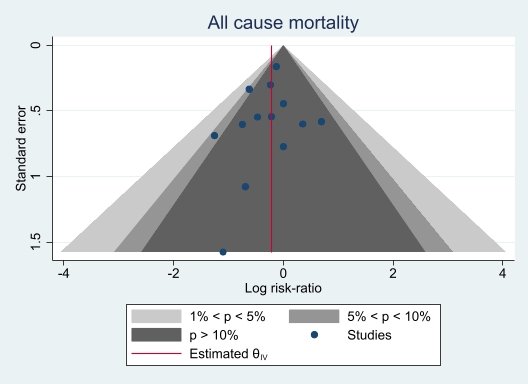


**Figure s5.** Contour funnel plot showing the effect of stem cell treatment on 28-day all-cause mortality as compared to SOC in patients with ARDS.


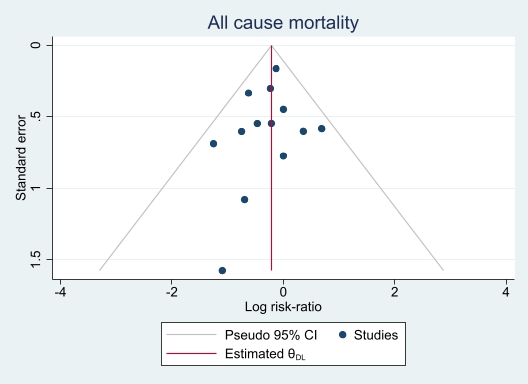


**Figure s6.** Funnel plot showing the effect of stem cell treatment on 28-day all-cause mortality as compared to SOC in patients with ARDS.


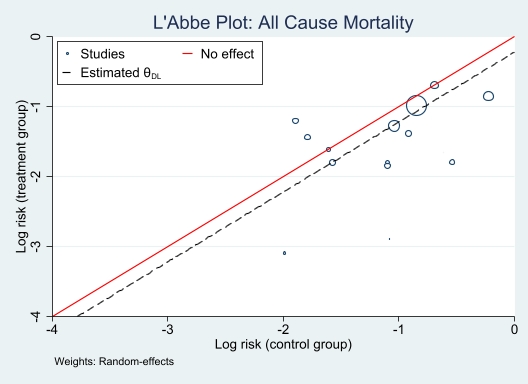


**Figure s7.** L’Abbe plot showing the effect of stem cell treatment on 28-day all-cause mortality as compared to SOC in patients with ARDS.


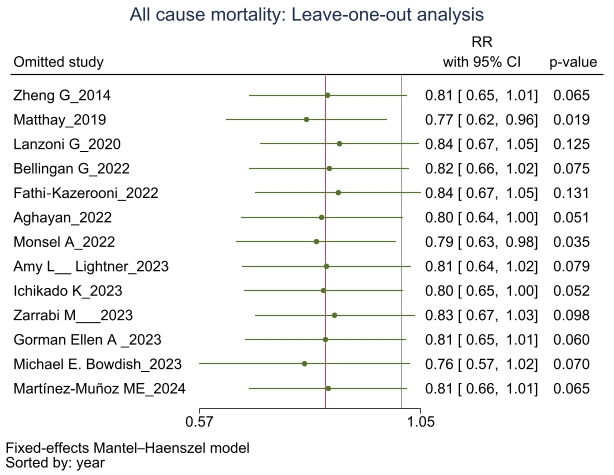


**Figure s8.** Leave-one-out sensitivity analysis showing the effect of stem cell treatment on 28-day all-cause mortality as compared to SOC in patients with ARDS.


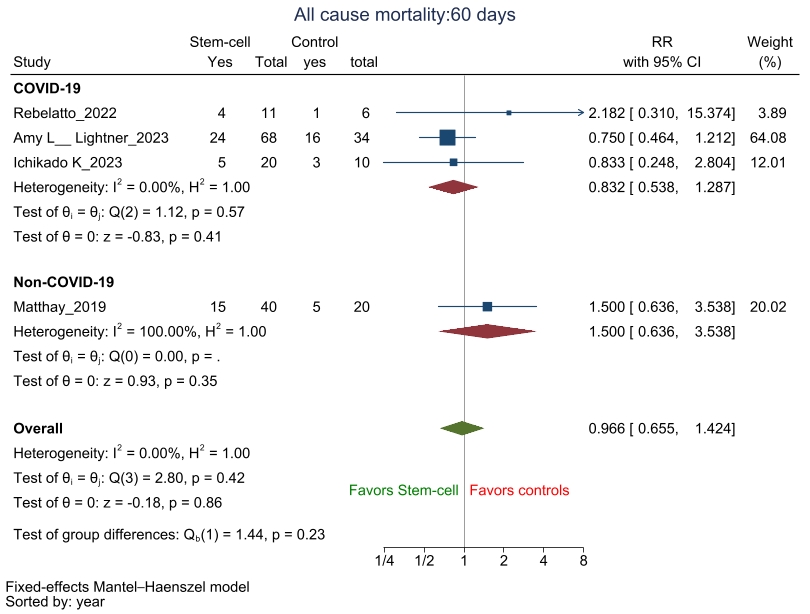


**Figure s9.** Sub-group analysis of the effect of stem cell treatment on 60-day all-cause mortality as compared to SOC in patients with ARDS.


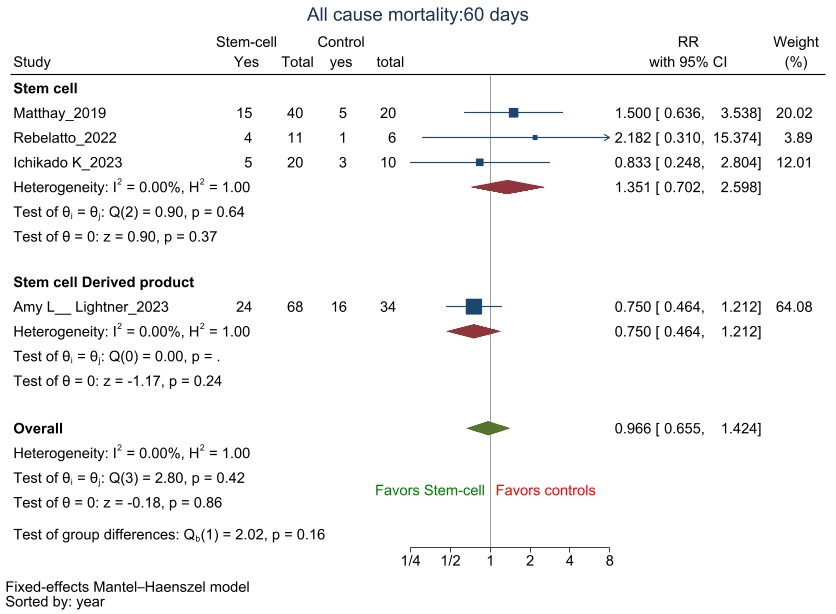


**Figure s10.** Sub-group analysis of the effect of stem cell treatment on 60-day all-cause mortality as compared to SOC in patients with ARDS based on source of stem cells.

**
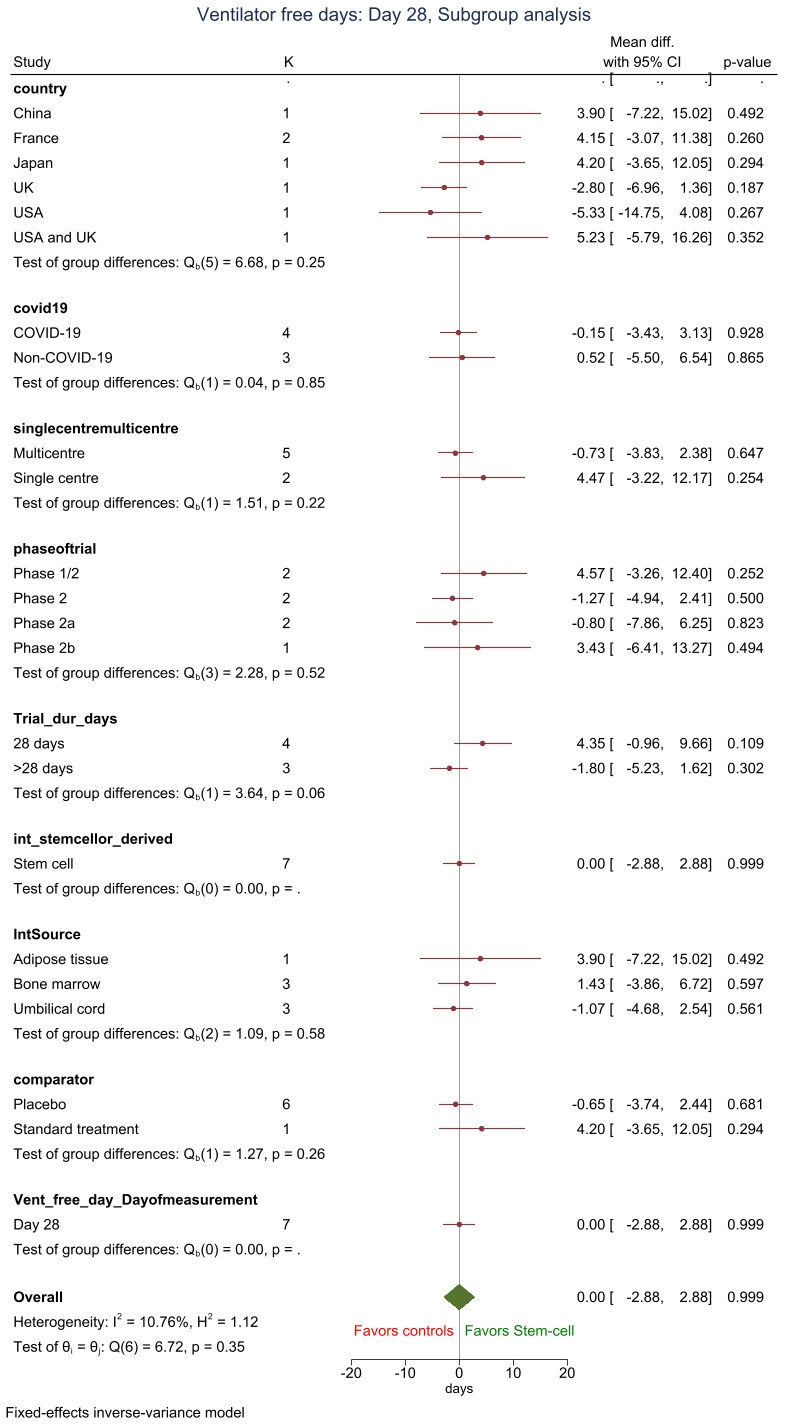
**

**Figure s11.** Sub-group analysis of the effect of stem cell treatment on ventilator-free days as compared to SOC in patients with ARDS.


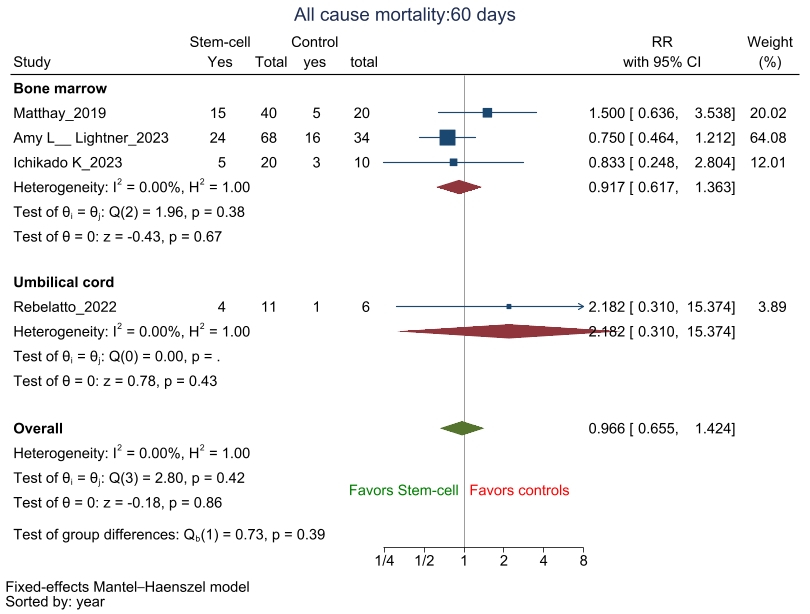


**Figure s12.** Sub-group analysis of the effect of stem cell treatment on 60-day all-cause mortality as compared to SOC in patients with ARDS based on type of stem cells.


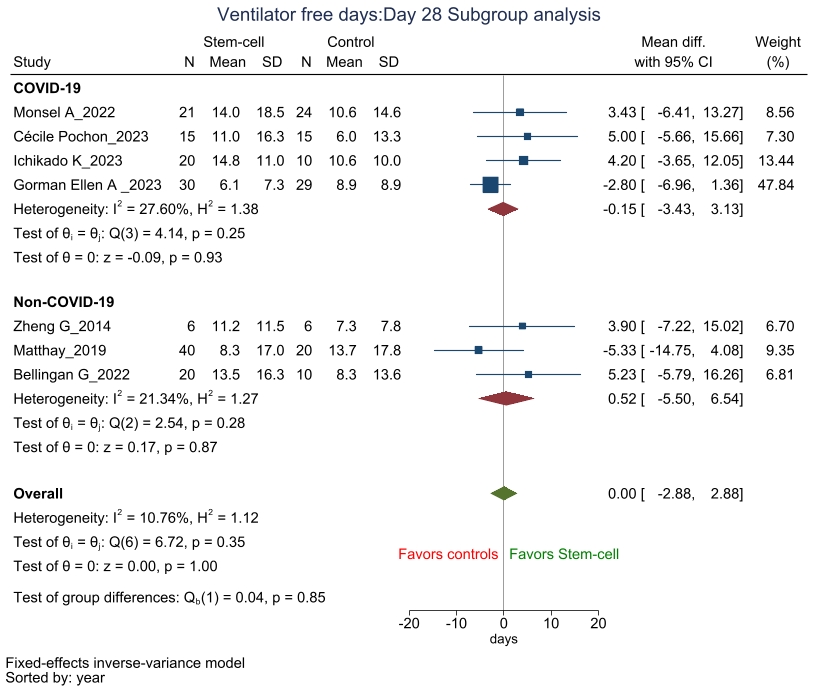


**Figure s13.** Sub-group analysis of the effect of stem cell treatment on ventilator-free days as compared to SOC in patients with ARDS based on etiology.


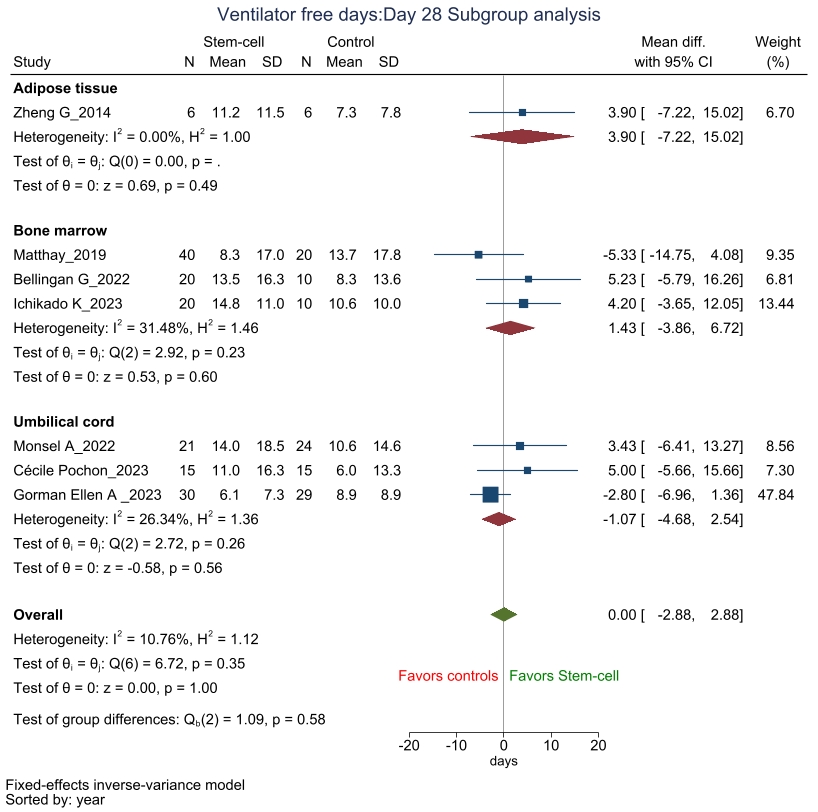


**Figure s14.** Sub-group analysis of the effect of stem cell treatment on ventilator-free days as compared to SOC in patients with ARDS based on source of stem cells.


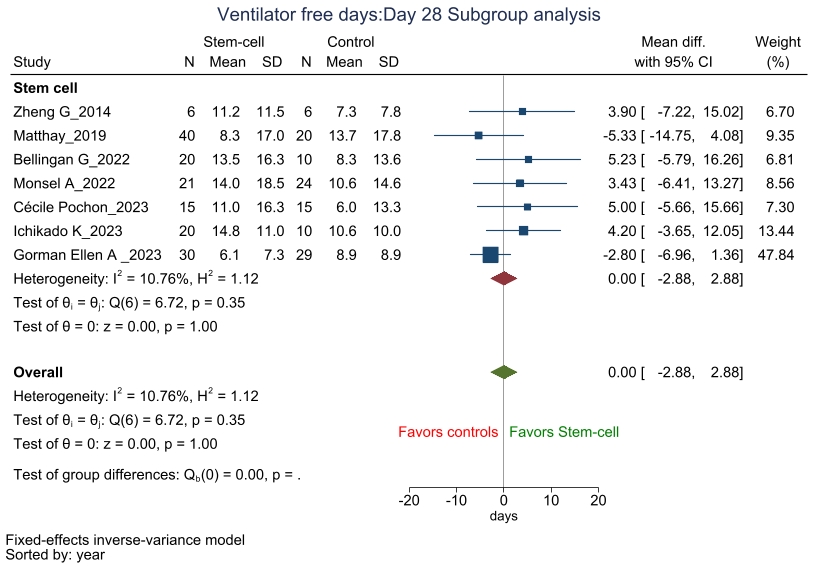


**Figure s15.** Sub-group analysis of the effect of stem cell treatment on ventilator-free days as compared to SOC in patients with ARDS based on type of stem cells.

**
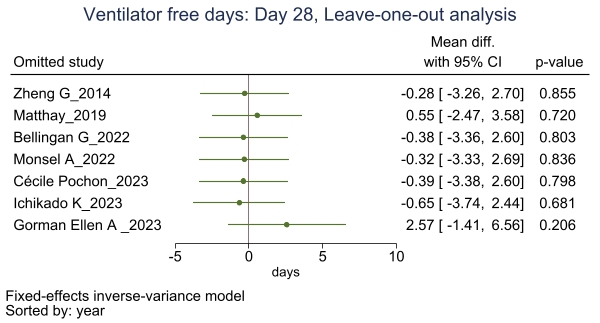
**

**Figure s16.** Leave-one-out sensitivity analysis showing the effect of stem cell treatment on ventilator-free days as compared to SOC in patients with ARDS.

**
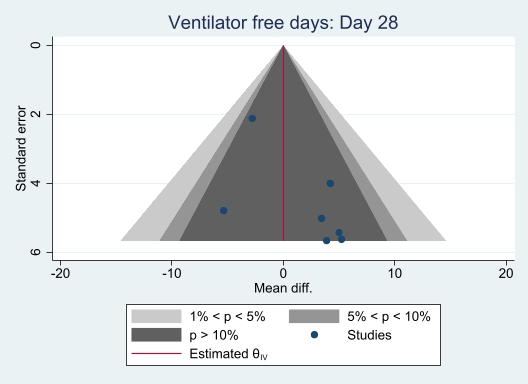
**

**Figure s17.** Contour funnel plot showing the effect of stem cell treatment on ventilator-free days as compared to SOC in patients with ARDS.

**
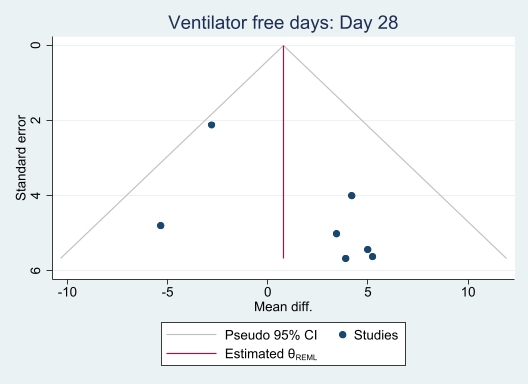
**

**Figure s18.** Funnel plot showing the effect of stem cell treatment on ventilator-free days as compared to SOC in patients with ARDS.


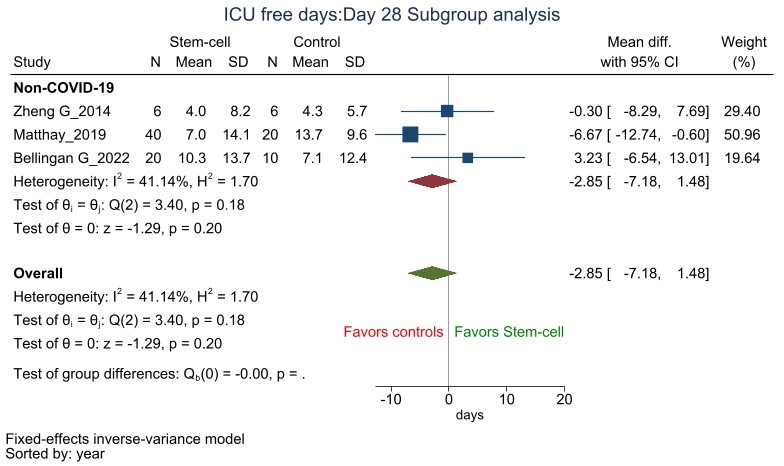


**Figure s19.** Sub-group analysis of the effect of stem cell treatment on ICU-free days as compared to SOC in patients with ARDS based on etiology.


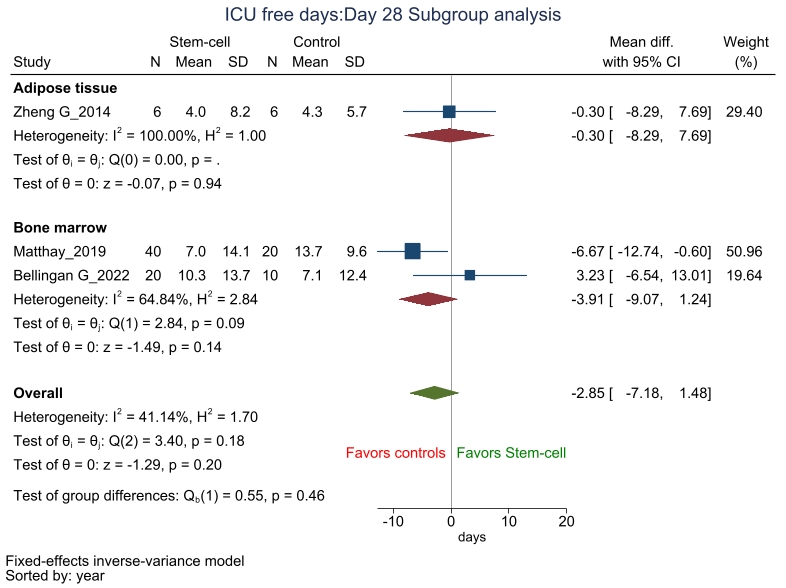


**Figure s20.** Sub-group analysis of the effect of stem cell treatment on ICU-free days as compared to SOC in patients with ARDS based on source of stem cells.


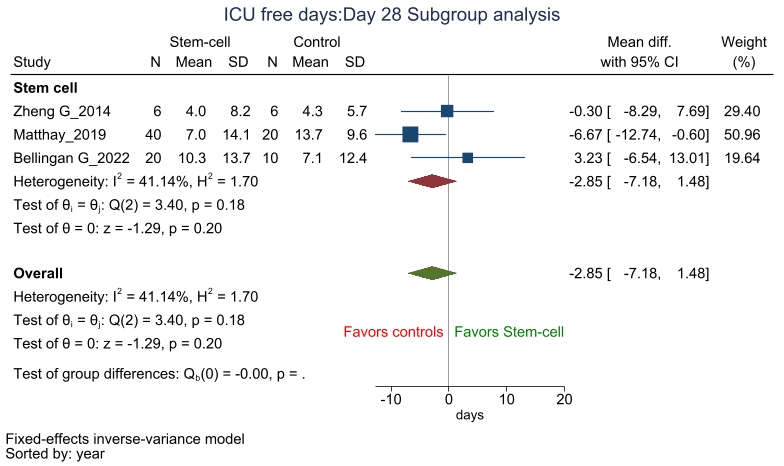


**Figure s21.** Sub-group analysis of the effect of stem cell treatment on ICU-free days as compared to SOC in patients with ARDS based on type of stem cells.


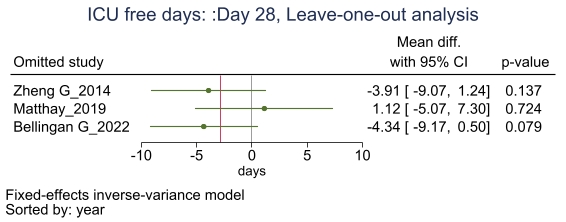


**Figure s22.** Leave-one-out sensitivity analysis showing the effect of stem cell treatment on ICU-free days as compared to SOC in patients with ARDS.


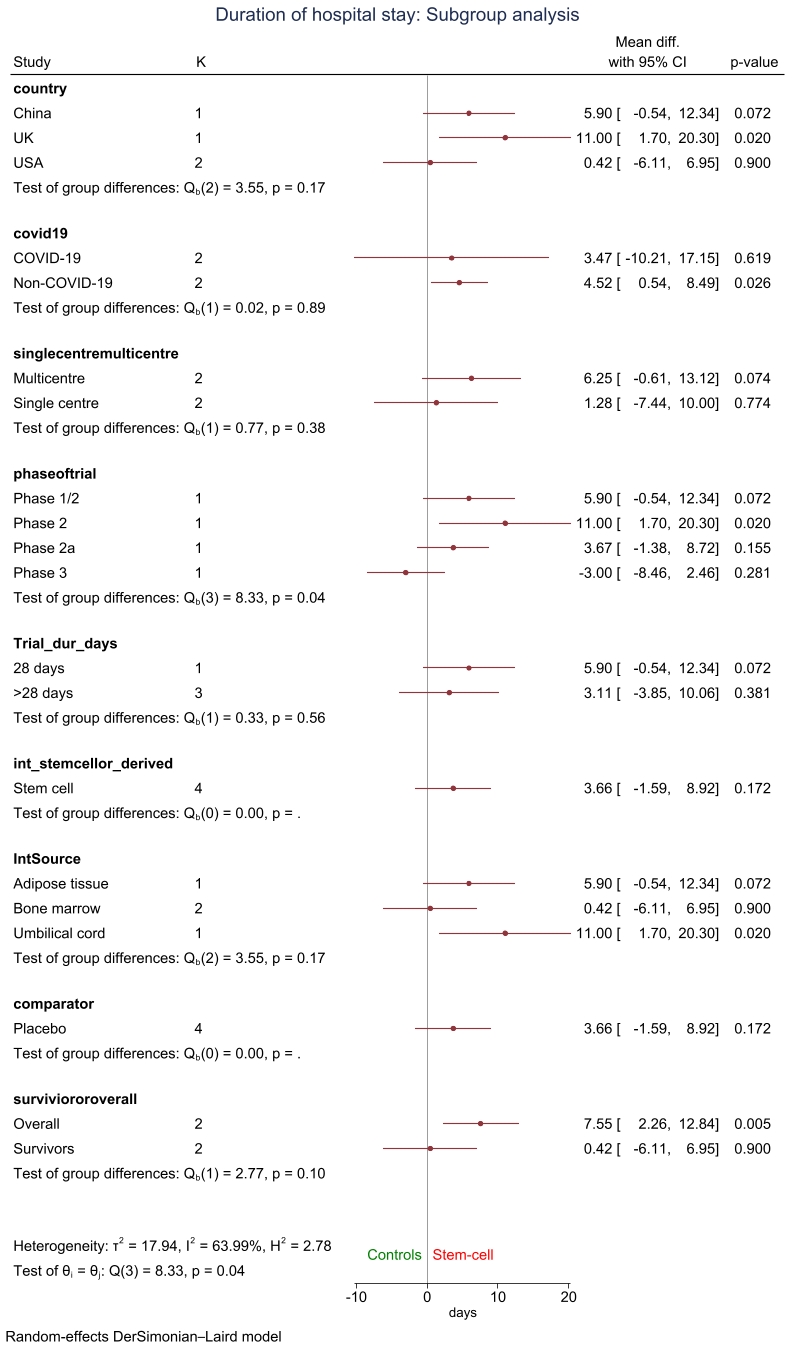


**Figure s23.** Sub-group analysis of the effect of stem cell treatment on the duration of hospitalization as compared to SOC in patients with ARDS.
