# Supplementary material for: Efficacy and safety of stem cell therapy vs. standard of care in patients diagnosed with acute respiratory distress syndrome: an updated systematic review and meta-analysis of randomized controlled trials
Source: Front Med (Lausanne). 2026 Jan 14;12:1674720. doi: 10.3389/fmed.2025.1674720 (PMC12847309; doi:10.3389/fmed.2025.1674720)
Supplement: Supplementary file 2 [file Data_Sheet_2.docx]

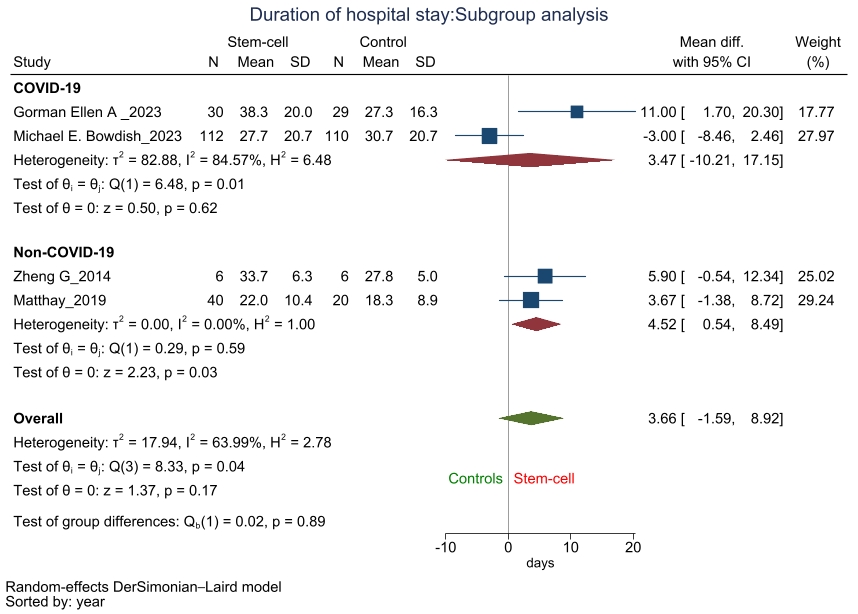


**Figure s24.** Sub-group analysis of the effect of stem cell treatment on the duration of hospitalization as compared to SOC in patients with ARDS based on etiology.


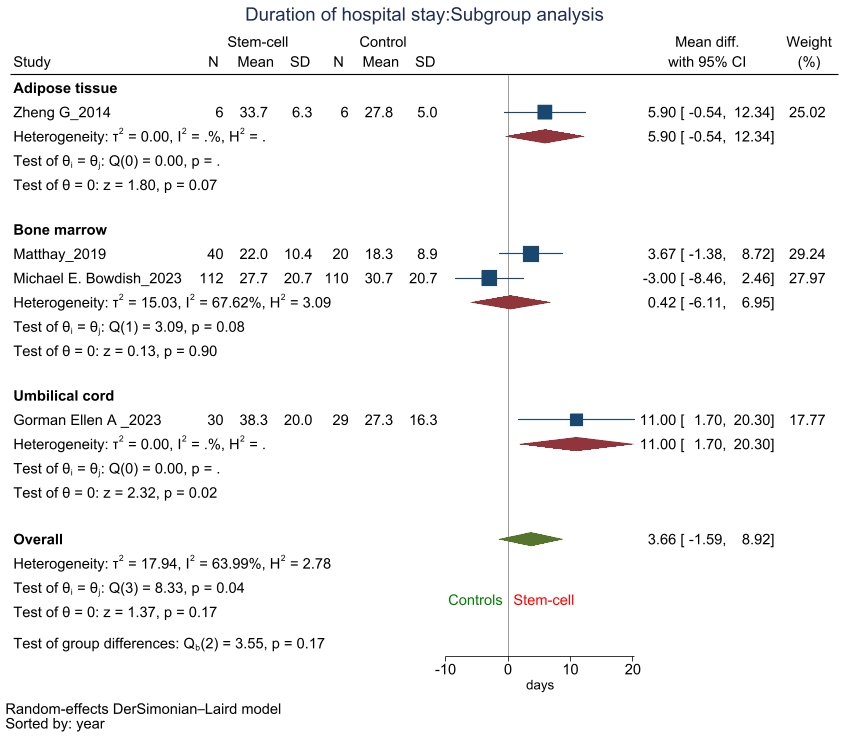


**Figure s25.** Sub-group analysis of the effect of stem cell treatment on the duration of hospitalization as compared to SOC in patients with ARDS based on source of stem cells.


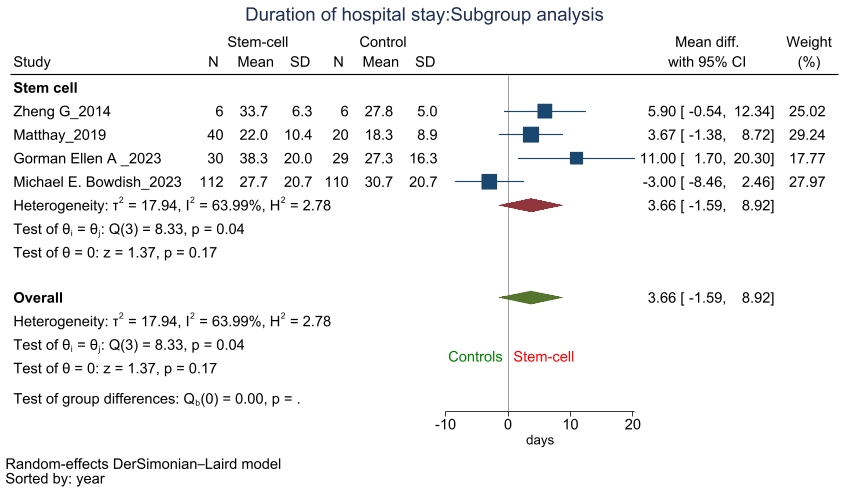


**Figure s26.** Sub-group analysis of the effect of stem cell treatment on the duration of hospitalization as compared to SOC in patients with ARDS based on type of stem cells.


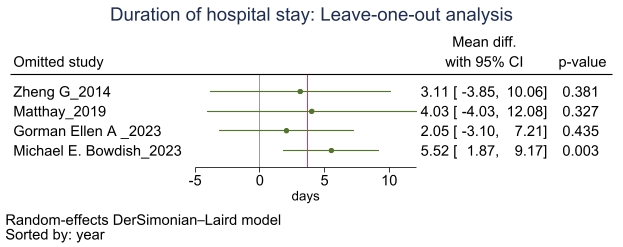


**Figure s27.** Leave-one-out sensitivity analysis showing the effect of stem cell treatment on the duration of hospitalization as compared to SOC in patients with ARDS.


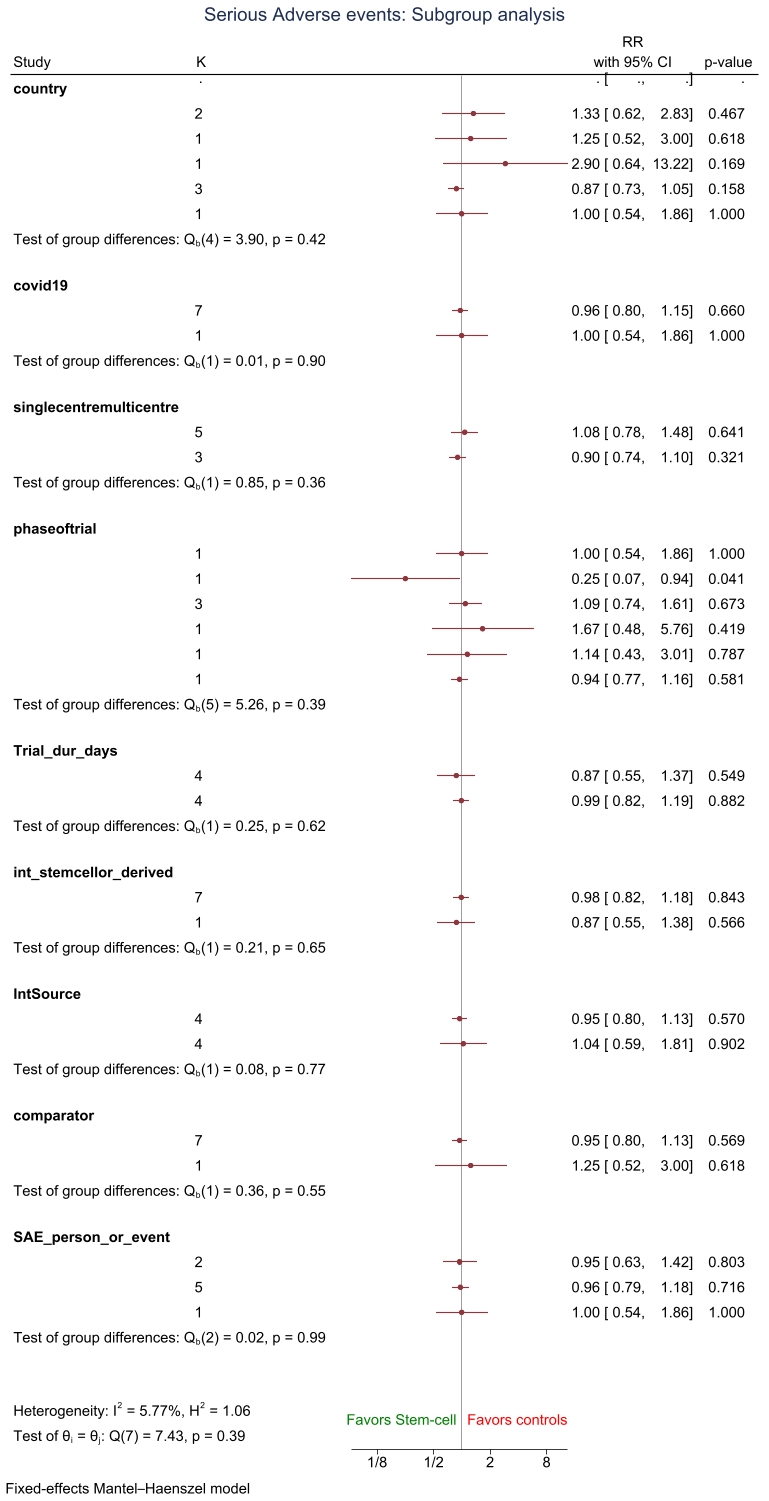


**Figure s28.** Sub-group analysis of the effect of stem cell treatment on SAE as compared to SOC in patients with ARDS.


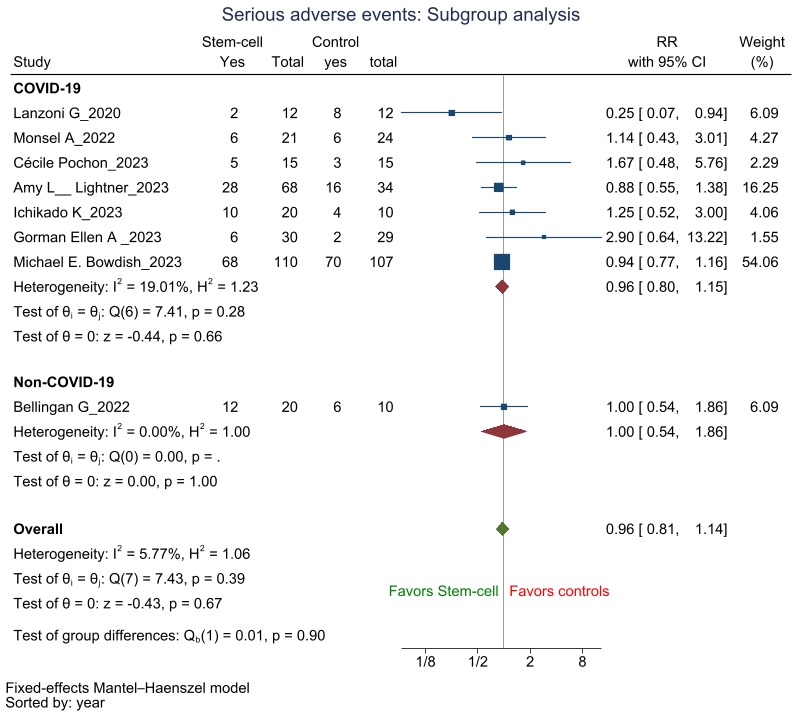


**Figure s29.** Sub-group analysis of the effect of stem cell treatment on SAE as compared to SOC in patients with ARDS based on etiology.


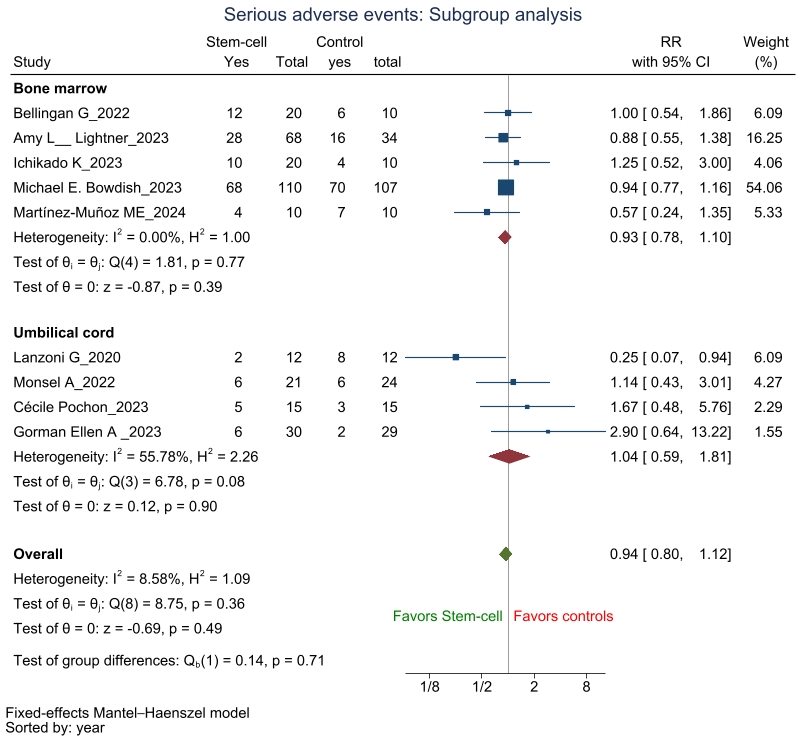


**Figure s30.** Sub-group analysis of the effect of stem cell treatment on SAE as compared to SOC in patients with ARDS based on source of stem cells.


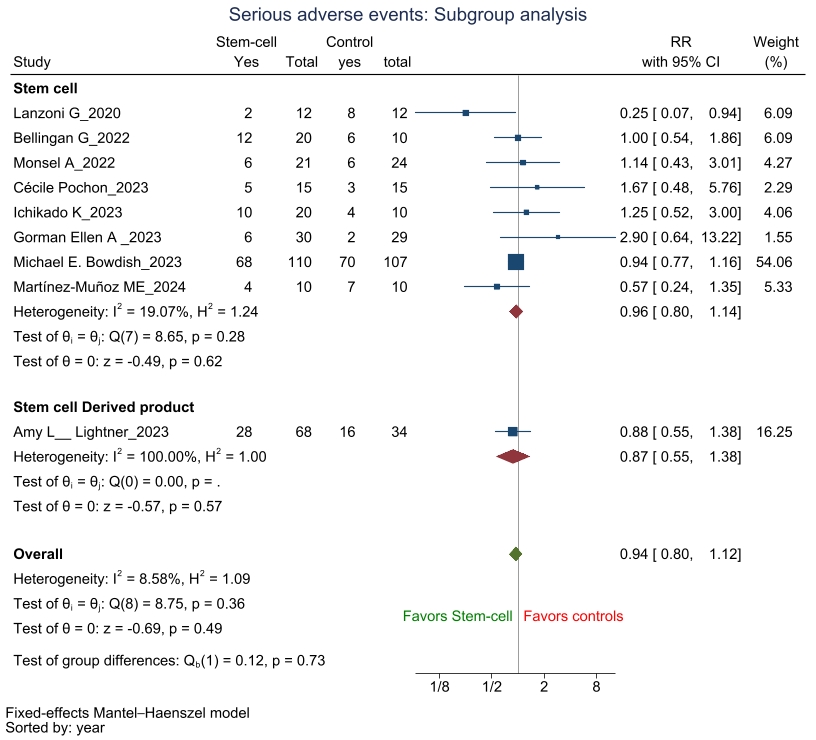


**Figure s31.** Sub-group analysis of the effect of stem cell treatment on SAE as compared to SOC in patients with ARDS based on type of stem cells.

.


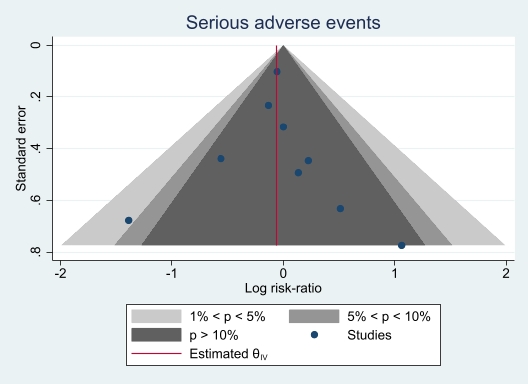


**Figure s32.** Contour funnel plot showing the effect of stem cell treatment on SAE as compared to SOC in patients with ARDS.


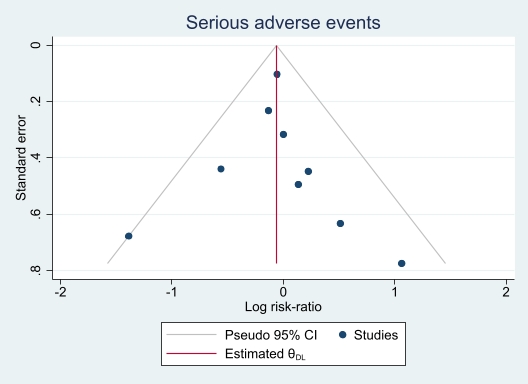


**Figure s33.** Funnel plot showing the effect of stem cell treatment on SAE as compared to SOC in patients with ARDS.


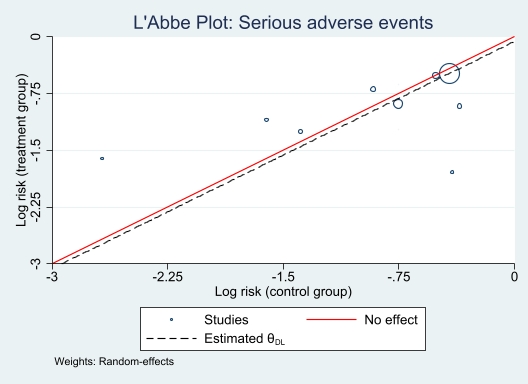


**Figure s34.** L’Abbe plot showing the effect of stem cell treatment on SAE as compared to SOC in patients with ARDS.


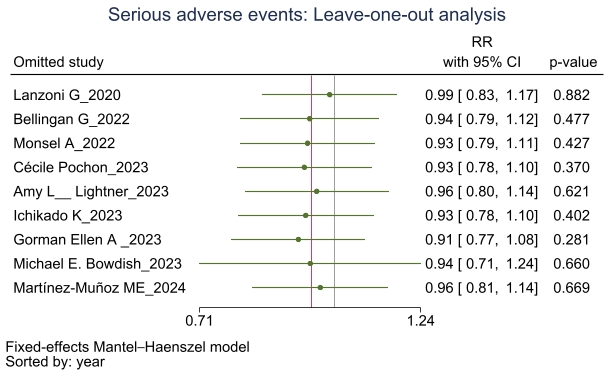


**Figure s35.** Leave-one-out sensitivity analysis showing the effect of stem cell treatment on SAE as compared to SOC in patients with ARDS.


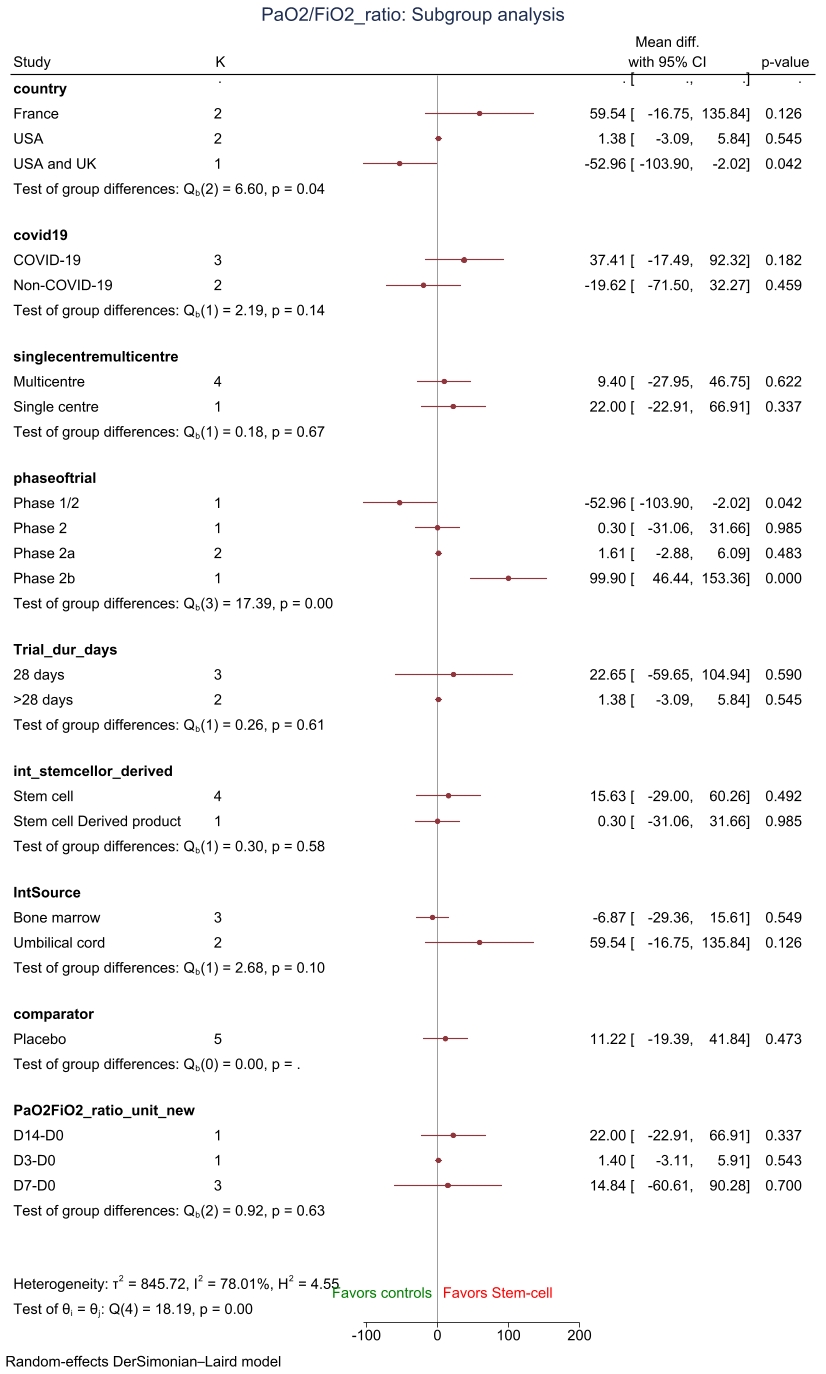


**Figure s36.** Sub-group analysis of the effect of stem cell treatment on PaO_2_/FiO_2_ ratio as compared to SOC in patients with ARDS.


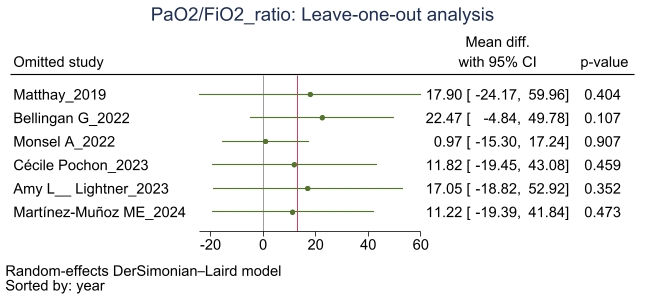


**Figure s37.** Leave-one-out sensitivity analysis showing the effect of stem cell treatment on PaO_2_/FiO_2_ ratio as compared to SOC in patients with ARDS.

**
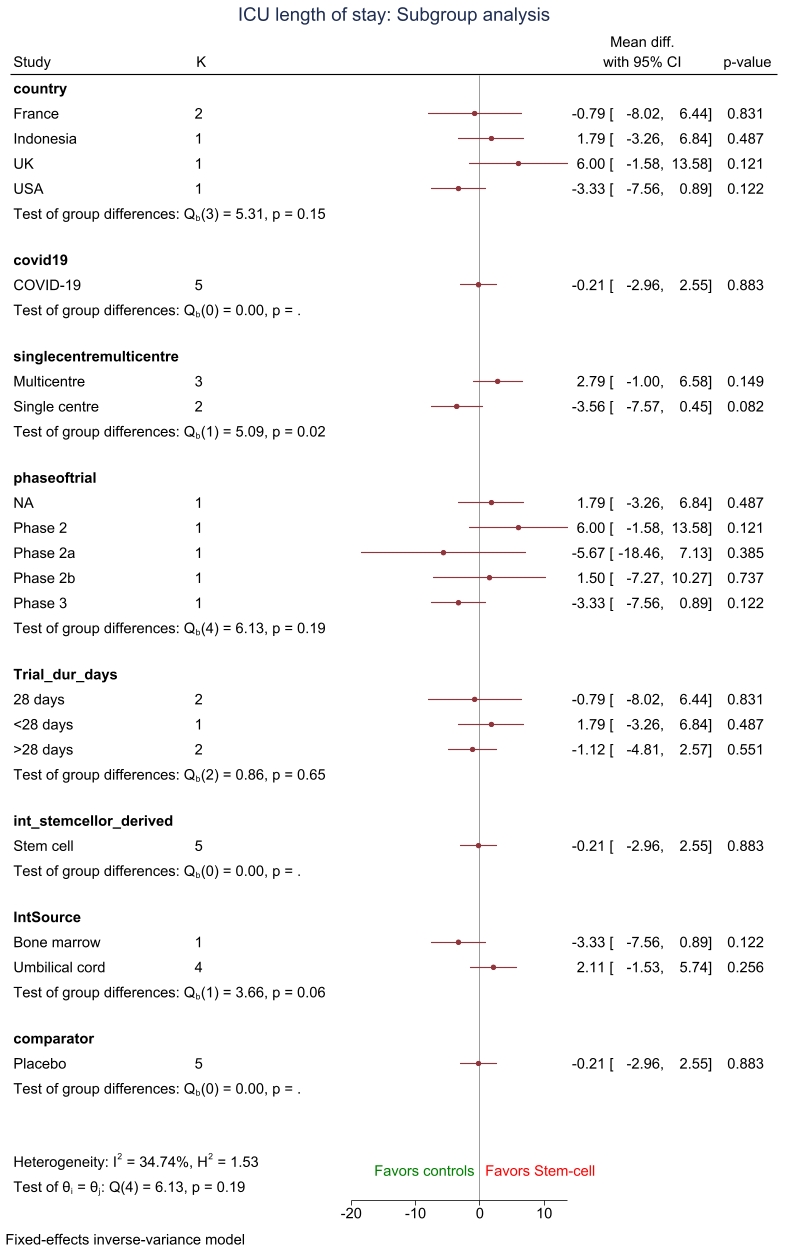
**

**Figure s38.** Sub-group analysis of the effect of stem cell treatment on ICU length of stay as compared to SOC in patients with ARDS.


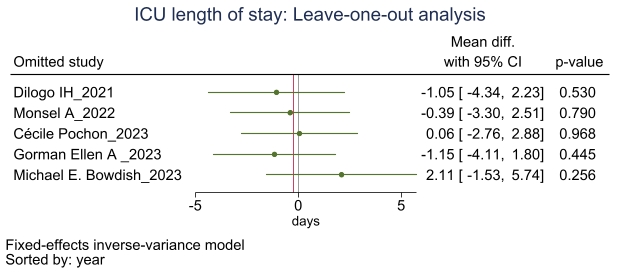


**Figure s39.** Leave-one-out sensitivity analysis showing the effect of stem cell treatment on ICU length of stay as compared to SOC in patients with ARDS.

**
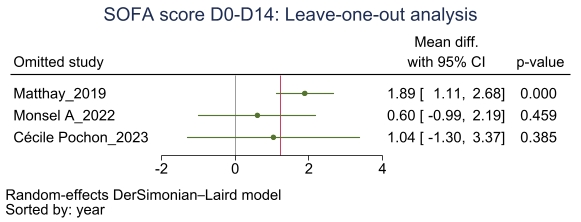
**

**Figure s40.** Leave-one-out sensitivity analysis showing the effect of stem cell treatment on SOFA score as compared to SOC in patients with ARDS.


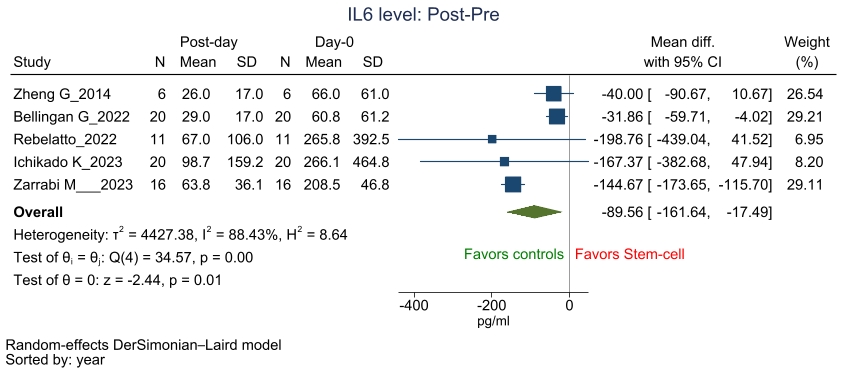


**Figure s41.** Effect of stem cell treatment on IL-6 level (post vs. pre comparison) as compared to SOC in patients with ARDS.


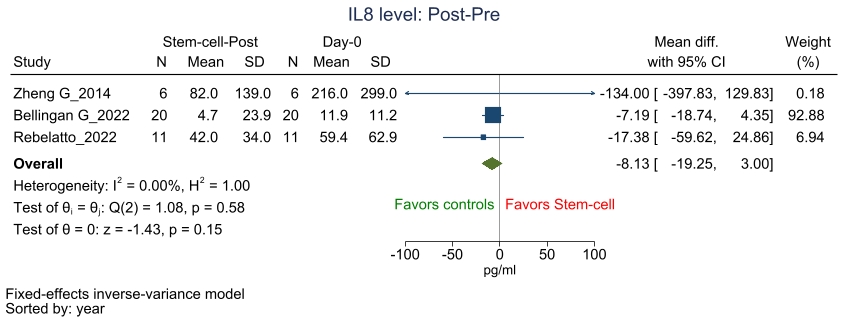


**Figure s42.** Effect of stem cell treatment on IL-8 level (post vs. pre comparison) as compared to SOC in patients with ARDS.

**
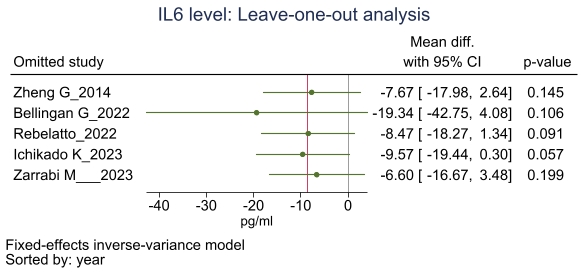
**

**Figure s43.** Leave-one-out sensitivity analysis showing the effect of stem cell treatment on IL-6 level as compared to SOC in patients with ARDS.

**
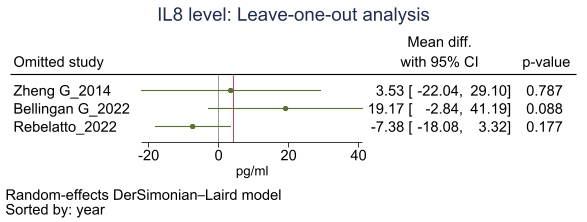
**

**Figure s44.** Leave-one-out sensitivity analysis showing the effect of stem cell treatment on IL-8 level as compared to SOC in patients with ARDS.

**
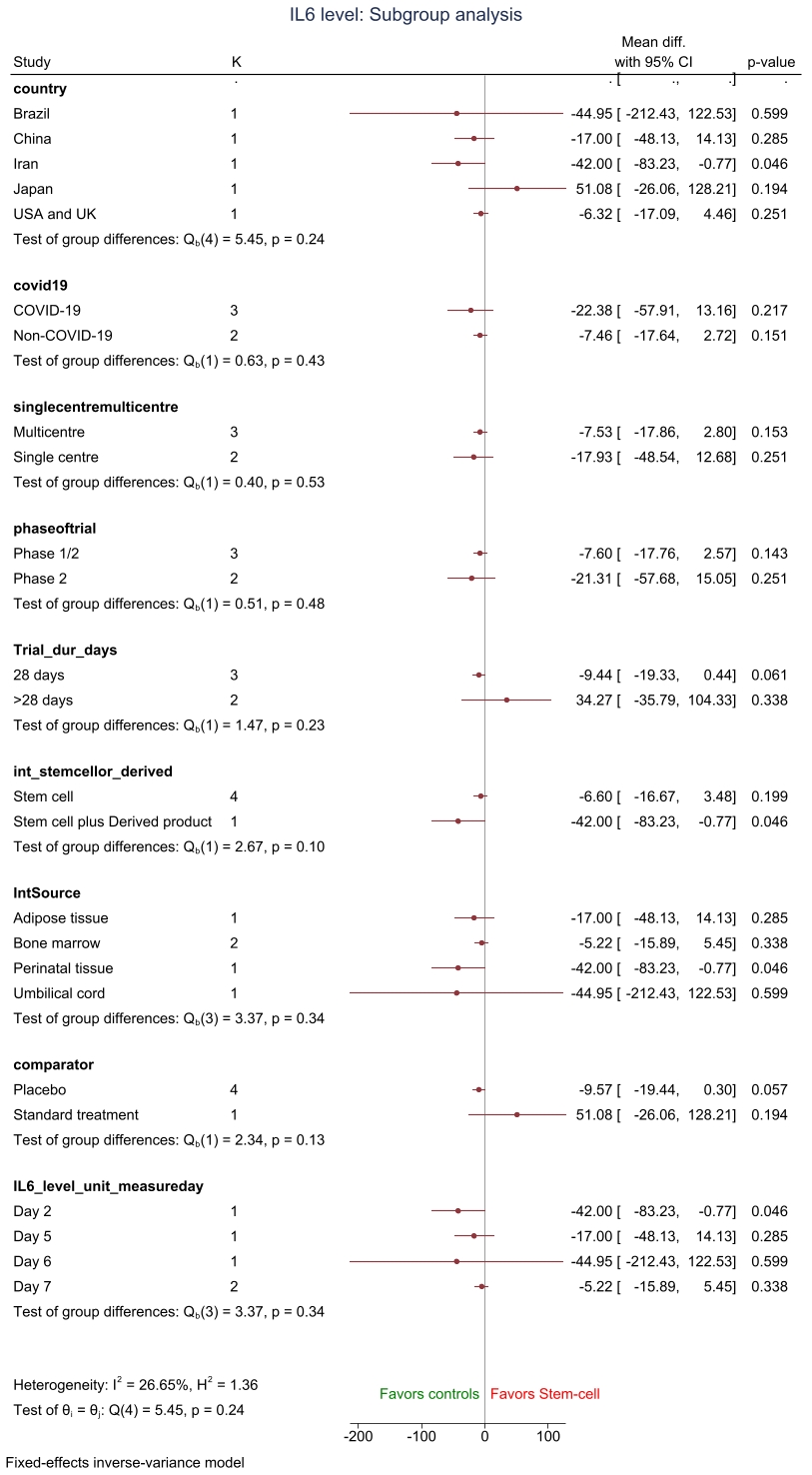
**

**Figure s45.** Sub-group analysis of the effect of stem cell treatment on IL-6 level as compared to SOC in patients with ARDS based on source of stem cells.


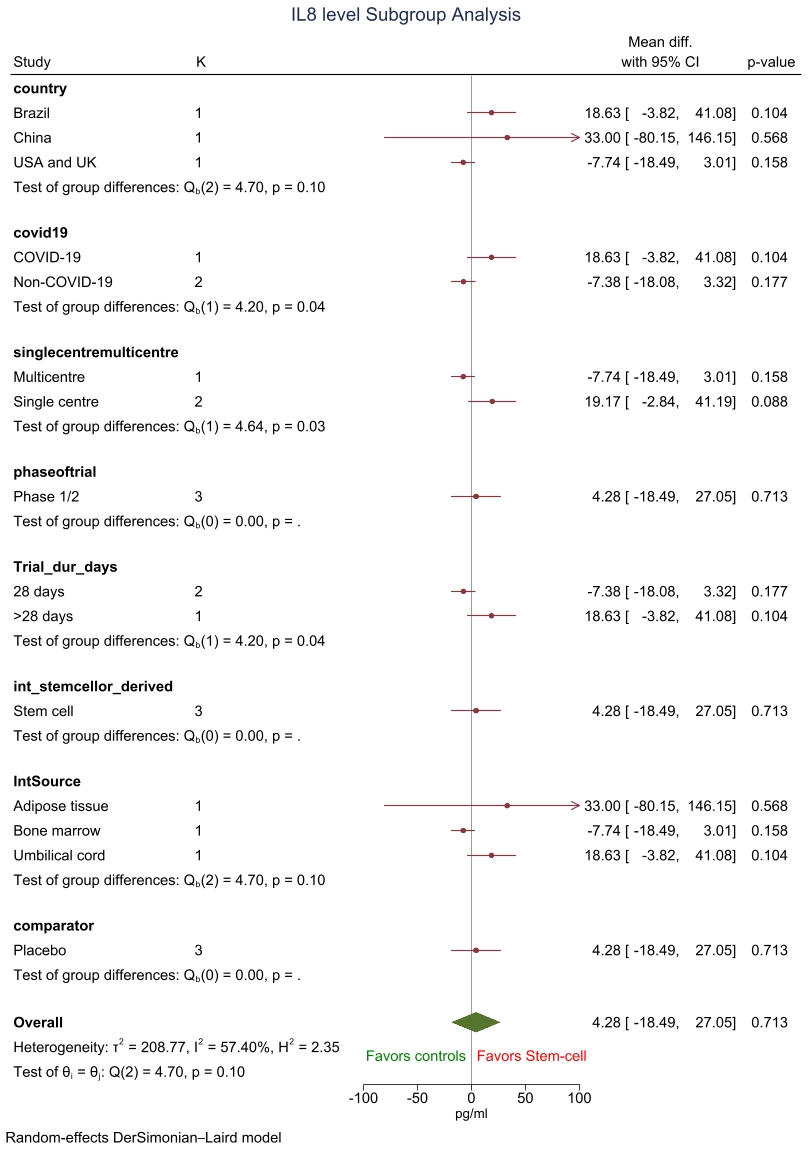


**Figure s46.** Sub-group analysis of the effect of stem cell treatment on IL-6 level as compared to SOC in patients with ARDS based on source of stem cells.
