# Supplementary material for: Efficacy and safety of stem cell therapy vs. standard of care in patients diagnosed with acute respiratory distress syndrome: an updated systematic review and meta-analysis of randomized controlled trials
Source: Front Med (Lausanne). 2026 Jan 14;12:1674720. doi: 10.3389/fmed.2025.1674720 (PMC12847309; doi:10.3389/fmed.2025.1674720)
Supplement: Supplementary file 3 [file Table_1.docx]

**Table s1.** Search term and search strategy in various databases.

| **Domain** | | **Pubmed Search terms /strategy** |
| --- | --- | --- |
| P-ARDS | | (acute respiratory distress syndrome) OR (acute respiration distress syndrome) OR ARDS OR (acute lung injury) OR “ALI” OR (adult respiration distress) OR (lung shock) OR (posttraumatic lung failure) OR (respiratory distress syndro*) OR (shock lung) |
| I- Stem-cells | | (stem cell*) OR "Stem cell treatment” OR “Mesenchymal Stem Cells” OR “Mesenchymal Stromal Cells” OR “MSC” OR “MSCs” OR “Induced pluripotent stem cells” OR “iPSc” OR (progenitor cells) OR (mother cell*) OR (Colony-Forming Unit*) OR (Colony Forming Unit*) OR mesenchymal OR (Bone Marrow Stromal Cell*) OR (Wharton Jelly Cell*) OR (Wharton's Jelly Cell*) OR teratocarcinoma OR (embryonal carcinoma Cell*) OR pluripotent OR multipotent OR totipotent OR (tumor initiating cell*) OR Hematopoietic OR Limbal OR “mESC” OR “mESCs” OR “hESCs” OR (Erythroid Precursor) OR CFU-E OR “CFU E” OR CFU-Es OR (Erythroid Progenitor Cell*) OR Erythropoietic OR (Burst-Forming Units) OR (Burst Forming Units) OR BFU-E OR (BFU E) OR BFU-Es |
| C | | -nil- |
| O | | -nil- |
| S-trials | | randomi* OR RCT OR non-randomi* OR (non randomi*) OR Placebo OR trial OR (clinical trial) OR feasibility OR safety |
|  | | P&I&S |
|  | | P&I&S AND (("2024/10/29"[Date - Create] : "3000"[Date - Create])) Sort by: Publication Date |
| **Domain** | **Embase Search terms /strategy** | |
| P-ARDS | 'adult respiratory distress syndrome'/exp OR 'ards' OR 'acute respiration distress syndrome' OR 'acute respiratory disease syndrome' OR 'acute respiratory distress syndrome' OR 'adult respiration distress' OR 'adult respiratory distress' OR 'adult respiratory distress syndrome' OR 'lung shock' OR 'posttraumatic lung failure' OR 'posttraumatic pulmonary insufficiency' OR 'respiratory distress syndrome, acute' OR 'respiratory distress syndrome, adult' OR 'respiratory distress, adult' OR 'shock lung' OR 'acute lung injury'/exp OR 'acute lung injury' OR `ali` | |
| I- Stem-cells | 'mesenchyme cell'/exp OR 'mesenchyma cell' OR 'mesenchymal cell' OR 'mesenchyme cell' OR 'mesenchymal stem cell'/exp OR 'mesenchymal progenitor cell' OR 'mesenchymal stem cell' OR 'mesenchymal stem cells' OR 'stem cell, mesenchymal' OR msc OR 'pluripotent stem cell'/exp OR 'pluripotent cell' OR 'pluripotent precursor cell' OR 'pluripotent progenitor cell' OR 'pluripotent stem cell' OR 'pluripotent stem cells' OR 'stem cell'/exp OR 'cell, stem' OR 'precursor cell' OR 'progenitor cell' OR 'stem cell' OR 'stem cells' OR 'induced pluripotent stem cell'/exp OR 'ips cell' OR 'induced pluripotent stem cell' OR 'induced pluripotent stem cells' OR 'mother cell'/exp OR 'colony forming cell'/exp OR 'cell, colony forming' OR 'colony forming cell' OR 'colony forming unit'/exp OR 'cfu' OR 'colony form unit' OR 'colony forming ability' OR 'colony forming capacity' OR 'colony forming unit' OR 'bone marrow stroma cell'/exp OR 'bone marrow stroma cell' OR 'bone marrow stromal cell' OR 'wharton jelly'/exp OR 'wharton jelly' OR 'mesenchymal stroma cell'/exp OR 'wharton jelly cell' OR 'mesenchymal stroma cell' OR 'mesenchymal stroma cells' OR 'mesenchymal stromal cell' OR 'mesenchymal stromal cells' OR 'teratocarcinoma cell line'/exp OR 'terato-carcinoma cell line' OR 'terato-carcinoma-derived cell line' OR 'teratocarcinoma cell line' OR 'teratocarcinoma-derived cell line' OR 'teratoid carcinoma cell line' OR 'teratomatous carcinoma cell line' OR 'embryonal carcinoma stem cell'/exp OR 'embryocarcinoma cell' OR 'embryocarcinoma cells' OR 'embryonal carcinoma cell' OR 'embryonal carcinoma cells' OR 'embryonal carcinoma stem cell' OR 'embryonal carcinoma stem cells' OR 'embryonic carcinoma cell' OR 'embryonic carcinoma cells' OR 'embryonic carcinoma stem cell' OR 'embryonic carcinoma stem cells' OR 'teratocarcinoma cell' OR 'teratocarcinoma cells' OR 'teratocarcinoma stem cell' OR 'teratocarcinoma stem cells' OR 'multipotent stem cell'/exp OR 'multipotent cell' OR 'multipotent precursor cell' OR 'multipotent progenitor cell' OR 'multipotent stem cell' OR 'multipotent stem cells' OR 'totipotent stem cell'/exp OR 'totipotent cell' OR 'totipotent precursor cell' OR 'totipotent progenitor cell' OR 'totipotent stem cell' OR 'totipotent stem cells' OR 'cancer stem cell'/exp OR 'cancer stem cell' OR 'cancer stem cells' OR 'neoplastic stem cell' OR 'neoplastic stem cells' OR 'stem cell, tumor' OR 'stem cell, tumour' OR 'tumor stem cell' OR 'tumor stem cells' OR 'tumor-initiating cell' OR 'tumor-initiating cells' OR 'tumour stem cell' OR 'tumour stem cells' OR 'hematopoietic stem cell'/exp OR 'bone marrow stem cell' OR 'haematopoietic precursor cell' OR 'haematopoietic progenitor cell' OR 'haematopoietic stem cell' OR 'haematopoietic stem cells' OR 'hematocytopoietic stem cell' OR 'hematopoietic precursor cell' OR 'hematopoietic progenitor cell' OR 'hematopoietic stem cell' OR 'hematopoietic stem cells' OR 'hemocytopoietic stem cell' OR 'hemopoietic stem cell' OR 'limbal stem cell transplantation'/exp OR 'limbal stem cell transplantation' OR 'limbal stem cell'/exp OR 'corneal epithelial stem cell' OR 'corneal limbal epithelial stem cell' OR 'limbal stem cell' OR 'limbal stem cells' OR mesc OR 'human embryonic stem cell'/exp OR 'cell, human embryonic stem' OR 'cells, human embryonic stem' OR 'hesc' OR 'hescs' OR 'human es cell' OR 'human es cells' OR 'human embryonic stem cell' OR 'human embryonic stem cells' OR 'stem cell, human embryonic' OR 'stem cells, human embryonic' OR 'colony forming unit e'/exp OR 'cfu e' OR 'colony forming unit e' OR 'erythroid precursor cell'/exp OR 'erythroid precursor' OR 'erythroid precursor cell' OR 'erythroid precursor cells' OR 'erythroid precursors' OR 'erythroid progenitor' OR 'erythroid progenitor cell' OR 'erythroid progenitor cells' OR 'erythroid progenitors' OR 'erythroid stem cell' OR 'erythroid stem cells' OR 'erythropoietic precursor' OR 'erythropoietic precursors' OR 'erythropoietic progenitor' OR 'erythropoietic progenitor cell' OR 'erythropoietic progenitors' OR 'erythropoietic stem cell' OR 'erythropoietic stem cells' OR 'precursor cell, erythroid' OR 'burst forming unit e'/exp OR 'bfu e' OR 'burst forming unit e' OR 'burst forming unit erythroid' OR 'burst-forming unit erythroid progenitor' OR 'erythroid burst forming unit' | |
| C | -nil- | |
| O | -nil- | |
| S-trials | 'randomized controlled trial'/exp OR 'controlled trial, randomized' OR 'randomised controlled study' OR 'randomised controlled trial' OR 'randomized controlled study' OR 'randomized controlled trial' OR 'trial, randomized controlled' OR 'clinical trial'/exp OR 'clinical drug trial' OR 'clinical trial' OR 'major clinical trial' OR 'trial, clinical' OR feasibility OR 'safety' | |
|  | P&I&S | |
|  | #1 AND #2 AND #3 AND [29-10-2024]/sd NOT [10-29-2024]/sd | |
| **Domain** | **Web of Science Search terms /strategy** | |
| P-ARDS | (((((((((TS=(acute respiratory distress syndrome )) OR TS=(acute respiration distress syndrome)) OR TS=(ARDS)) OR TS=(acute lung injury)) OR TS=(ALI)) OR TS=(adult respiration distress)) OR TS=(lung shock)) OR TS=(posttraumatic lung failure)) OR TS=(respiratory distress syndro*)) OR TS=(shock lung) and Preprint Citation Index (Exclude – Database) | |
| I- Stem-cells | ((((((((((((((((((((((((((((((((((((TS=(stem cell*)) OR TS=(Stem cell treatment)) OR TS=("Mesenchymal Stem Cells” )) OR TS=(“Mesenchymal Stromal Cells”)) OR TS=(MSC)) OR TS=(MSCs)) OR TS=(“Induced pluripotent stem cells” )) OR TS=(“iPSc” )) OR TS=(progenitor cells)) OR TS=(mother cell*)) OR TS=(Colony-Forming Unit*)) OR TS=(mesenchymal )) OR TS=(Bone Marrow Stromal Cell*)) OR TS=(Wharton Jelly Cell*)) OR TS=(Wharton's Jelly Cell*)) OR TS=(teratocarcinoma)) OR TS=(embryonal carcinoma Cell*)) OR TS=(pluripotent )) OR TS=(multipotent )) OR TS=(totipotent )) OR TS=(tumor initiating cell*)) OR TS=(Hematopoietic)) OR TS=(Limbal )) OR TS=(“mESC” )) OR TS=(“mESCs”)) OR TS=(“hESCs” )) OR TS=((Erythroid Precursor) )) OR TS=(CFU-E )) OR TS=(“CFU E” )) OR TS=(CFU-Es )) OR TS=((Erythroid Progenitor Cell*) )) OR TS=(Erythropoietic)) OR TS=((Burst-Forming Units))) OR TS=((Burst Forming Units) )) OR TS=(BFU-E )) OR TS=((BFU E) )) OR TS=(BFU-Es) and Preprint Citation Index (Exclude – Database) | |
| S-trials | ((((((TS=(randomi*)) OR TS=(RCT )) OR TS=(non-randomi* )) OR TS=((non randomi*) )) OR TS=(Placebo)) OR TS=(trial )) OR TS=((clinical trial)) and Preprint Citation Index (Exclude – Database) | |
|  | P&I&S | |
| **Domain** | **Cochrane Search terms /strategy** | |
| P-ARDS | ("acute respiratory distress syndrome" OR "acute respiration distress syndrome" OR ARDS OR "acute lung injury" OR "ALI" OR "adult respiration distress" OR "lung shock" OR "posttraumatic lung failure" OR "respiratory distress syndrome" OR "respiratory distress syndromes" OR "shock lung") | |
| I- Stem-cells | ("stem cell" OR "stem cells" OR "Stem cell treatment" OR "Mesenchymal Stem Cells" OR "Mesenchymal Stromal Cells" OR "MSC" OR "MSCs" OR "Induced pluripotent stem cells" OR "iPSc" OR "progenitor cells" OR "mother cell" OR "mother cells" OR "Colony-Forming Unit" OR "Colony-Forming Units" OR "Colony Forming Unit" OR "Colony Forming Units" OR "mesenchymal" OR "Bone Marrow Stromal Cell" OR "Bone Marrow Stromal Cells" OR "Wharton Jelly Cell" OR "Wharton Jelly Cells" OR "Wharton's Jelly Cell" OR "Wharton's Jelly Cells" OR "teratocarcinoma" OR "embryonal carcinoma Cell" OR "embryonal carcinoma Cells" OR "pluripotent" OR "multipotent" OR "totipotent" OR "tumor initiating cell" OR "tumor initiating cells" OR "Hematopoietic" OR "Limbal" OR "mESC" OR "mESCs" OR "hESCs" OR "Erythroid Precursor" OR "CFU-E" OR "CFU E" OR "CFU-Es" OR "Erythroid Progenitor Cell" OR "Erythroid Progenitor Cells" OR "Erythropoietic" OR "Burst-Forming Units" OR "Burst Forming Units" OR "BFU-E" OR "BFU E" OR "BFU-Es") | |
| C | -nil- | |
| O | -nil- | |
| S-trials | ("randomised" OR "randomized" OR "RCT" OR "non-randomised" OR "non-randomized" OR "non randomised" OR "non randomized" OR "Placebo" OR "trial" OR "clinical trial" OR "feasibility" OR "safety") | |
|  | P&I&S | |
|  | P&I&S AND (("2024/10/29"[Date - Create] : "3000"[Date - Create])) Sort by: Publication Date | |
